# Significance of Polymers with “Allyl” Functionality in Biomedicine: An Emerging Class of Functional Polymers

**DOI:** 10.3390/pharmaceutics14040798

**Published:** 2022-04-06

**Authors:** Mijanur Rahman, Aliaa Ali, Erica Sjöholm, Sebastian Soindinsalo, Carl-Eric Wilén, Kuldeep Kumar Bansal, Jessica M. Rosenholm

**Affiliations:** 1 Pharmaceutical Sciences Laboratory, Faculty of Science and Engineering, Åbo Akademi University, BioCity, Tykistökatu 6A, 20520 Turku, Finland; mijanur.rahman@abo.fi (M.R.); aliaa.a.ali@utu.fi (A.A.); erica.sjoholm@abo.fi (E.S.); sebastian.soidinsalo@abo.fi (S.S.); 2 Laboratory of Molecular Science and Engineering, Åbo Akademi University, Aurum, Henrikinkatu 2, 20500 Turku, Finland; cwilen@abo.fi

**Keywords:** allyl-terminated polymers, post-synthesis functionalization, drug delivery, controlled drug release, functional polymers

## Abstract

In recent years, polymer-based advanced drug delivery and tissue engineering have grown and expanded steadily. At present, most of the polymeric research has focused on improving existing polymers or developing new biomaterials with tunable properties. Polymers with free functional groups offer the diverse characteristics needed for optimal tissue regeneration and controlled drug delivery. Allyl-terminated polymers, characterized by the presence of a double bond, are a unique class of polymers. These polymers allow the insertion of a broad diversity of architectures and functionalities via different chemical reactions. In this review article, we shed light on various synthesis methodologies utilized for generating allyl-terminated polymers, macromonomers, and polymer precursors, as well as their post-synthesis modifications. In addition, the biomedical applications of these polymers reported in the literature, such as targeted and controlled drug delivery, improvement i aqueous solubility and stability of drugs, tissue engineering, and antimicrobial coatings, are summarized.

## 1. Introduction

A vast variability of polymers originating from natural and synthetic sources are currently utilized in various biomedical applications, especially in drug delivery and tissue engineering. Among the different polymers, polyesters [1,2,3], polyethers [4,5,6,7], polyanhydrides [8,9,10], poly(ester-anhydride)s [11,12], and polysaccharides [5,13,14] are the most common groups of polymers widely applied in the biomedical sector. Additionally, polystyrene, polyethyleneimine, and polyurethane-based polymers are also investigated in similar contexts [15,16,17,18,19,20]. Both non-biodegradable and biodegradable polymers are used in drug delivery [21,22,23]. Each group of polymers or individual polymers holds great potential and, in some cases, unique properties to be used as carriers for various types of drugs and in other biomedical applications. Biodegradable polymers are extensively used therein due to their biodegradability and biocompatibility. Natural and synthetic biodegradable polymers are extensively explored in the biomedical field due to the presence of unstable functional groups in their backbone [14,24,25,26,27]. These groups undergo hydrolytic or enzymatic degradation in vivo and are thus excreted from the body without inducing any undesired effects.

Chitosan, polylactide (PLA), poly(ε-caprolactone) (PCL), poly(carboxyphenoxy hexane-sebacic acid), poly(ethylene glycol), polymethyl methacrylate (PMMA), and poly(propylene glycol) (PPG) are examples of polymers investigated for and used in pharmaceutical applications. However, almost all of these polymers lack free functional or pendant groups for further chemical and/or physical modifications.

A polymer matrix should possess a number of desirable properties, such as hydrophobicity, stability, toughness, flexibility, solubility in organic solvents, a low melting point, and degradability in a constant manner over time in physiological conditions, to be considered as a suitable matrix for drug delivery [28]. However, native forms of polymers can be seen to suffer from lack of one, or several, of these properties. For example, the native form of PLA is a biodegradable, biocompatible, and bioabsorbable biopolymer derived from agricultural sources and possesses eminent transparency, good mechanical properties, and can be processed using traditional processing technologies to make various forms of PLA products with desired shapes. PLA’s outstanding properties, such as renewability, biodegradability, biocompatibility, and low carbon emission during production etc., renders it one of the most popular polymers in biomaterials research. Nonetheless, it has a lack of functionality or pendant groups for further modifications, poor surface adhesive nature due to a fairly hydrophobic surface, insufficient barrier performance to oxygen (O_2_), carbon dioxide (CO_2_) and nitrogen (N_2_), all of which limit the applicability of PLA to many biomedical applications, such as cell adhesion and drug delivery. Due to its poor surface properties, PLA-based drug delivery carriers often display poor drug loading, which ultimately makes them less attractive for drug delivery application, compared to hydrophilic polymers. Similarly, neat PCL is another biodegradable, biocompatible, nontoxic, and bioresorbable aliphatic polyester, like PLA. It has been extensively investigated for the development of implantable biomaterials, contraceptive devices, fixation devices, wound dressings, and controlled release and targeted drug delivery. It can be degraded by chemical and enzymatic hydrolysis of its ester linkages under physiological conditions (such as in the human body) and many different environments (e.g., in pure fungal cultures, compost, active sludge, and soil). However, PCL displays poor surface wettability and cell attachment compared to hydrophilic materials, due to its hydrophobic surface nature. In addition, it exhibits relatively slow degradation (2–4 years) depending on the molecular weight [29,30] and inferior mechanical properties compared to other medical polyesters, which limit the uses of PCL to non-load bearing and slowly resorbable scaffolds. Generally, polymers are modified to overcome these limitations and the modification process involves the incorporation of new specific functionality in the polymer main chains, or as pendant groups, or by altering the surface functionality, and are brought about by copolymerization reactions, post-reaction modifications, by reactions with the existing functional groups or by blending with other polymers. However, several amphiphilic biodegradable copolymers of PLA, PCL, and PGA described in the literature failed to preserve the balance between the responsiveness of the carrier and their stability in physiological conditions, because they do not possess appropriate functionalities to be used for controlled drug release triggered by external stimuli (heat [31], light [32], pH [33], or redox conditions [34]).

With the advent of the modern technological era, special functions or multifunctional and adaptive polymer materials are required, where parent forms of the routinely utilized natural and synthetic polymers might not meet these specifications. For instance, an appropriate functional group along the polymer chain is a prerequisite to facilitate the conjugation of drugs [35], biological ligands, or medical imaging probes. Allyl-terminated or allyl-functionalized polymers have recently found application in many fields, owing to the tailoring of their properties as per the requirements, simply by tweaking the free functional groups on unsaturated hydrocarbons. Over the past few decades, allyl functional groups containing polymers have been the focus of intensive research for their extensive application, primarily, in allyl resins and electronic and electrical engineering industries because of their exceptional physical and electrical properties. Their uses within the biomedical fields are still limited. However, the scenario has been changing in the recent years. Allyl-terminated polymers or macromonomers are synthesized by using an allyl-functionalized initiator or monomer, or by modifications with an allyl compound. To date, several synthesis processes have successfully been applied for the preparations of allyl-terminated polymers, and several post-modification strategies have been effectively utilized. The most common synthesis methods of allyl-terminated polymers are ring-opening polymerization (ROP) [36,37,38,39,40,41,42], anionic ring-opening polymerization [43], reversible addition-fragmentation chain transfer (RAFT) polymerization [44], atom transfer radical polymerization (ATRP) [45,46,47], and condensation reactions [48,49,50]. The thiol–ene photoinitiated, or thermal polymerization, method is the method most vastly applied for the post-functionalization of allyl (ene) polymers. The thiol–ene reaction can be performed together with living polymerization, such as ROP [38,40,42,51], ring-opening metathesis polymerization (ROMP) [52,53], cationic polymerization [54,55], or controlled radical polymerization [56]. Thiol-ene click-reaction, epoxidation [40,42,57], bromination [40], dihydroxylation [57], reduction, oxidation, photo-induced crosslinking, grafting, and other addition reactions are commonly performed techniques for post-modification of allyl groups in polymers. These polymers are utilized in the delivery of drugs and bioactive agents [36,43,51,58,59,60], bacterial therapy [61], antimicrobial coatings [37], dental cavity restoration (thiol-ene formulations) [62,63,64], UV-cured coatings [65], artificial oxygen and protein encapsulation [43], allyl resins and oligomer applications [66,67,68,69].

To the best of our knowledge, no relevant review articles are available in the literature describing the synthesis techniques, post-modification, and potential biomedical applications of polymers with allyl functionality. Therefore, the aim of this review is to bridge the gap by highlighting the recent progress within functional polymers derived from “allyl” functionality and their utility in biomedical and related applications, particularly in advanced drug delivery.

## 2. Synthesis, Post-Modifications and Applications of “Allyl”-Terminated Polymers

### 2.1. Polyesters

**Figure 1:**
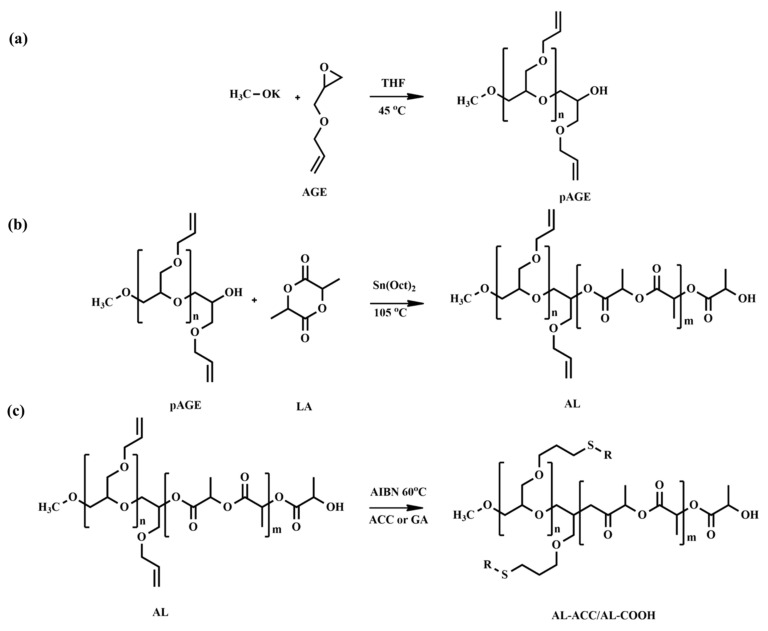
The scheme of synthesis of the pAGE-PLA copolymer. (**a**) Anionic polymerization of AGE, (**b**) coordination polymerization of LA initiated by pAGE, and (**c**) functionalization of AL with ACC or tGA (where R is ACC or -CH_2_-COOH moiety) as proposed by Kost et al. Reprinted with permission from [58]; published by Elsevier, 2021.

Several polyester-based copolymers with “allyl” functionality have been reported in the literature via ROP and post-functionalized by either click, epoxidation, or bromination reactions. For instance, Kost et al. [58] developed novel amorphous pH-tunable copolymers of lactide (LA) and allyl glycidyl ether (AGE) with functionalities for the efficient delivery of anti-cancer drugs. Poly(allyl glycidyl ether) (pAGE) was synthesized via anionic ROP and used as a macroinitiator for the ROP of LA to generate a copolymer, poly(allyl glycidyl ether-*co*-lactide) (AL), with free allyl functional groups in the backbone. To synthesize pAGE, the authors utilized anionic ROP instead of cationic ROP, as a later method produced a low molecular weight polymer. The synthesis process of AL copolymers was performed at a relatively low temperature (105 °C) to prevent the isomerization of unsaturated bonds on pAGE. The proton nuclear magnetic resonance (^1^H NMR) and size exclusion chromatography (SEC) analyses exhibited no significant isomerization of the double bond in the AL chains. The AL copolymers were not pH-responsive. Later, post-functionalization of the AL copolymers was performed by thiol-ene click reaction using thioglycolic acid (tGA) and N-acetyl-L-cysteine (ACC) to introduce pH-sensitive functional groups to the main chain pendants (Figure 1). The resulting post-functionalized copolymers with either ACC (AL ACC) or tGA (AL COOH) were found to be sensitive to pH changes as these compounds serve carboxyl or amino functionalities.

**Figure 2:**
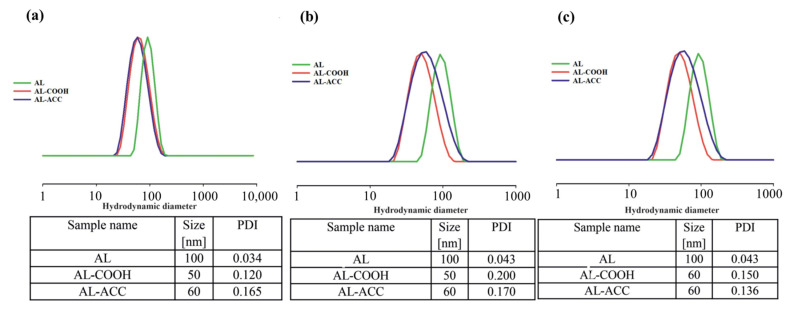
The size and dispersity of all prepared NPs in (**a**) water, (**b**) PBS, and (**c**) acetic acid buffer, as studied by Kost et al. Reprinted with permission from [58]; published by Elsevier, 2021.

The main focus of Kost et al.’s study [58] was to design biocompatible pH-responsive nanocarriers, which will not only deliver the anti-cancer drugs effectively to the desired targets, but also preserve the balance between the responsiveness of the nanocarriers and their stability in physiological conditions. With the aims of obtaining high drug encapsulation efficiency (EE), narrow size dispersity, and relatively small-size nanoparticles (NPs), the authors conducted a nanoprecipitation method to prepare blank and doxorubicin (DOX)-loaded NPs using neat AL copolymer, post-functionalized (AL ACC) and (AL COOH) copolymers as the matrix and poly(ethylene glycol) methyl ether-b-poly(D,L-lactide) (PEGME-*b*-PDLLA) as the non-ionic surfactant. The size and dispersity of all prepared NPs in water, PBS (pH = 7.0), and acetic acid buffer (pH = 5.0) are presented in Figure 2. The authors obtained larger NPs from non-functionalized AL copolymers (size 100 nm in all three media); this has been attributed to the rigidity of the pAGE chain due to the presence of unsaturated double bonds (which are known to prevent free rotation of molecules). On the other hand, functionalized copolymers produced NPs with a hydrodynamic size which was half that of their non-functionalized counterparts due to the increase in flexibility of the copolymer, with decrease in the number of allyl groups. Partial and full functionalization of allyl groups could be the factor in getting slightly smaller size and lower dispersity NPs from AL COOH, and slightly bigger and higher dispersity NPs from AL ACC copolymers, respectively. The authors assessed the change in size of NPs with respect to pH change by incubating NPs at 37 °C within 24 h and noticed no significant change in size of NPs in physiological and acidic conditions (pH = 7.0 and 5.0 respectively) with respect to the sizes in water. The stability of NPs with tGA and ACC in acidic pH 5.0 was accounted for by the formation of stable carboxylate moieties, negatively charged ions for both NPs with tGA and ACC, or the development of PEG corona on the surface of NPs with ACC. The authors’ explanation of the size of NPs in three different media was in agreement with studies in the literature [69,70,71,72,73]. However, the authors did not discuss the dispersity of the prepared NPs.

**Figure 3:**
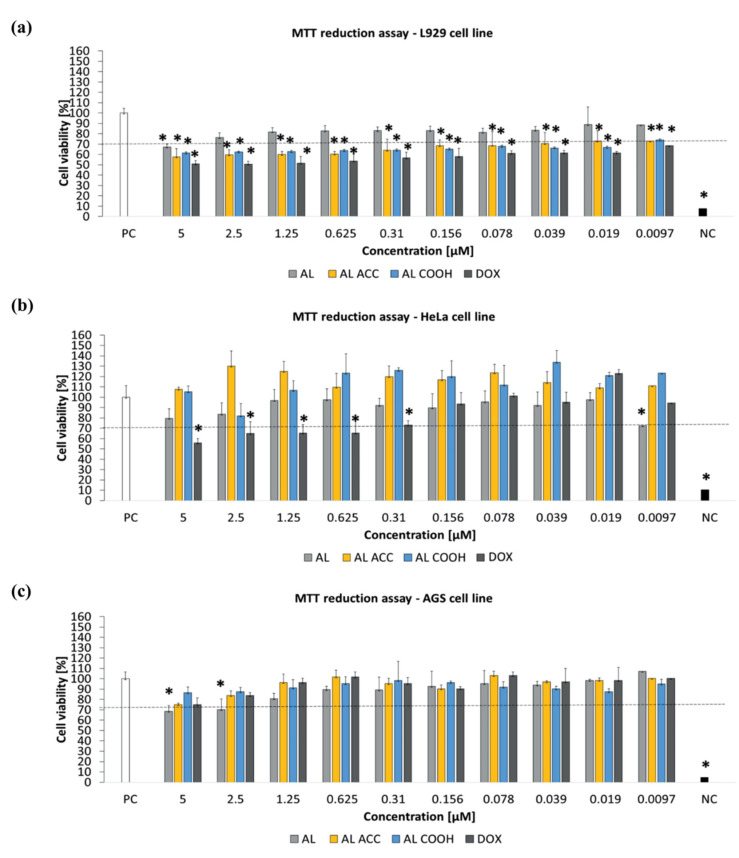
The cytotoxicity of blank NPs tested on (**a**) L929, (**b**) HeLa, and (**c**) AGS cell lines. Statistical significance: * *p* < 0.05; * untreated cells vs. cells treated with tested compounds as studied by Kost et al. Reprinted with permission from [58]; published by Elsevier, 2021.

The observed EE for DOX was ~36% and the release study suggested reduction in burst release due to the supramolecular interactions between polymeric pedant groups and DOX. Moreover, a slower release rate was observed at pH = 5.0 due to the strongest interaction between the AC ACC or AC COOH functionalities with DOX. In addition, ACC is well recognized for its high antioxidant properties; therefore, ACC containing nanocarriers can contribute to enhancing cancer treatment during cancer therapy. A microculture tetrazolium assay (MTT) assay was performed using murine fibroblast cell line (L929), human breast cancer cell line (HeLa), and human gastric adenocarcinoma cancer cell line (AGS) to evaluate the toxicity of blank and DOX-loaded NPs. A complete culture medium and 0.03% H_2_O_2_ were considered as a positive control of cell viability (100% viable cells) and a negative control of cell viability (100% dead inactive cells), correspondingly. The MTT assay revealed the non-toxicity of all blank NPs against all three tested cell lines after 24 h incubation at 37 °C (Figure 3). The authors stated that some tested compounds, e.g., DAPI (40, 6-diamino-2-phenylindole), might be subjected to Collagen type I secretion by endothelial or epithelial cells due to proregenerative activity, which eventually caused cell viability to pass over 100% in the case of the HeLa cell line. The concentration-dependent insignificant cytotoxicity of AL COOH and AL ACC NPs against the murine L929 fibroblasts observed in this study accounted for the strong interaction between tGA and ACC moieties of NPs and the cell membrane. This effect is supported by a study conducted by He [1].

**Figure 4:**
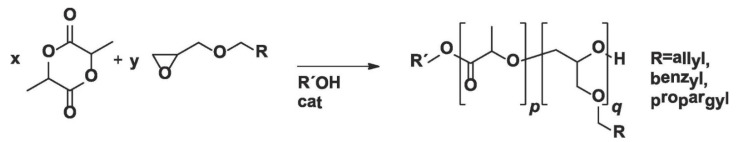
Introduction of allyl, benzyl, or propargyl functional group along a PLA chain via copolymerization of D,L-LA with glycidyl ethers as proposed by Pound-Lana et al. Reprinted with permission from [36]; published by Elsevier, 2017.

In a similar study, Pound-Lana et al. [36] synthesized LA copolymer with three glycidyl ethers (allyl, benzyl, and propargyl glycidyl ethers) containing either allyl or propargyl groups for further modifications via ROP using tin(II)octoate or 1,5,7-triazabicyclo [4.4.0]dec-5-ene (TBD) as catalyst (Figure 4). The authors observed that poly(lactide-co-allyl glycidyl ether) copolymer with 4.6 allyl groups per chain can be obtained by tin(II)octoate-catalyzed copolymerization of LA with AGE. However, the number of allyl groups per chain was reduced to less than one, and mostly PLA homopolymer was formed when TBD was used as the catalyst. The authors also found that a higher number of reactive groups per chain (up to 8.7 mole% of allyl functional groups) can be incorporated into the polymer backbone by increasing the feed ratio of AGE, but this comes with the expense of a decrease in molar mass of the copolymer. It was suggested that the decrease in molar mass was not due to systematic chain-termination but a chain transfer, as polydispersity values remained less than two. The authors proposed that, upon AGE incorporation, a chain is terminated to form a new polymer chain, which could be the main cause for the decrease in number average molecular weight (M_n_) with increasing AGE content in the feed. Nadeau et al. [41] reported similar observations, where a decrease in molar mass from 19,700 to 3300 g mol^−1^ was observed when they polymerized LA in the presence of AGE with feed content varying from 2 to 30 mol%, using tetraphenyltin as a catalyst.

**Figure 5:**
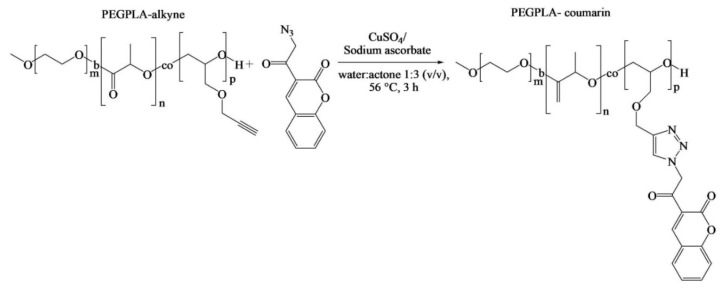
Reaction scheme for the preparation of alkyne-functional PEG-*b*-P(LA-*co*-PGE)-coumarin conjugate as described by Pound-Lana et al. Adapted with permission from [36]; published by Elsevier, 2017.

Although Pound-Lana [36] synthesized block copolymers containing allyl groups, only alkyne-functional PEG-*b*-P(LA-*co*-PGE) copolymer was used for the investigation of its application. A fluorescent azidocoumarin, 3-(α-azidoacetyl)coumarin, was conjugated to the polymer via copper-catalyzed Huisgen-1,3-dipolar cycloaddition (Figure 5). The authors proposed that the fluorescent labeled nanospheres could potentially act as drug carriers and imaging agents. However, the allyl-functional PEG-*b*-P(LA-*co*-AGE) copolymer synthesized in this study was not applied in fluorescent polymer nanosphere preparation; although, allyl groups of AGE unit in the copolymer could be tuned as per the desired application, or allow direct modification under “thiol-ene click” chemistry. Moreover, the facile modifications of AGE for grafting the part and inert character of the allyl group (in general alkene) under the polymerization conditions could be the principal advantage of using AGE.

**Figure 6:**
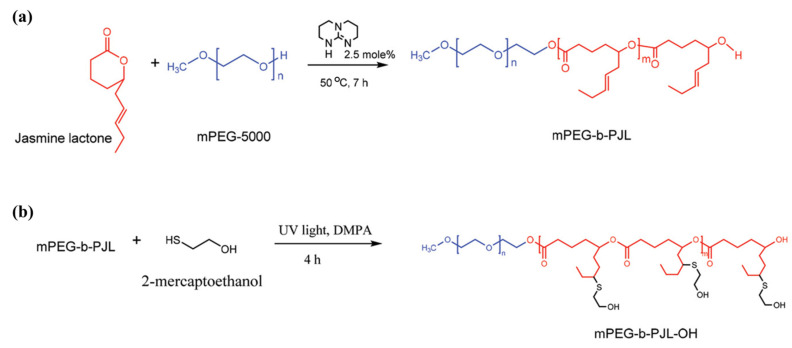
(**a**) Synthesis scheme of jasmine lactone block copolymer via ring-opening polymerization. (**b**) The block copolymer mPEG-*b*-PJL functionalization via UV-light induced thiol-ene click reaction to insert free alcohol groups (mPEG-*b*-PJL-OH) as represented by Bansal et al. [59].

Recently, Bansal et al. [59] took a smart approach and chose a commercially available starting lactone monomer that already contains free allyl groups on its chemical structure. The authors developed novel functional polymers from renewable feedstock jasmine lactone, using methoxy(polyethylene glycol) (mPEG) as initiator and TBD as catalyst. The post-functionalization of copolymer mPEG-*block*-poly(jasmine lactone), (mPEG-*b*-PJL) was then successfully demonstrated via UV assisted thiol-ene click chemistry to introduce hydroxyl, carboxyl, or amine functionality (Figure 6). Although the authors reported that the introduction of hydroxyl and carboxyl groups to the polymer using respective thiol was found to be very efficient, insertion of amine functionality did not yield 100% conversion.

Bansal et al. [59] utilized hydroxyl-terminated polymer (mPEG-*b*-PJL-OH) for the preparation of polymer-drug conjugates (PDCs) using DOX as a model drug via redox-responsive disulfide linkage, i.e., mPEG-*b*-PJL-S-S-DOX (PJL-DOX). The conjugation reaction followed a one-pot scheme; first, DOX reacted with DTPA, then, activation of the DTPA acid end group was followed by coupling of mPEG-*b*-PJL-OH. The resultant product, PJL-DOX, self-assembled into micelles with an average hydrodynamic size of *~*150 nm and demonstrated reduction-responsive DOX release. The authors observed that the PJL-DOX has the ability to stimulate the release of DOX due to the cleavage of disulfide linkage facilitated by glutathione (GSH). It was suggested that these stimuli-sensitive micelles could release the drug exclusively in cancerous cells in high amounts due to the presence of excessive GSH, and thus can mitigate the toxicity of cancerous drugs towards normal cells.

Le Devedec et al. [60] prepared a series of well-defined poly(valerolactone)-*co*-poly(allyl-δ-valerolactone) (PVL-*co*-PAVL) copolymers for use in drug delivery. The authors synthesized PVL-*co*-PAVL copolymers via metal-free ring-opening copolymerization of δ-Valerolactone (VL) and allyl δ-valerolactone (AVL) using TBD as a catalyst. In the synthesis process, the TBD catalyst was first placed in a flame-dried round two-neck Schlenk flask and dried under vacuum, and then anhydrous toluene and benzyl alcohol solvents were incorporated in the TBD-containing flask under argon and stirred for half an hour. Both VL and AVL monomers were distilled and transferred by cannulation into the reaction flask under positive pressure of argon. The polymerization reaction was conducted at room temperature for 6 h under argon atmosphere. After the polymerization was completed, the resulting slurry solution of copolymers was precipitated into cold methanol, and then re-dissolved in tetrahydrofuran (THF) and re-precipitated to the mixture of hexane/ethyl ether (3:7). The characteristics of copolymers are given in Table 1.

**Figure 7:**
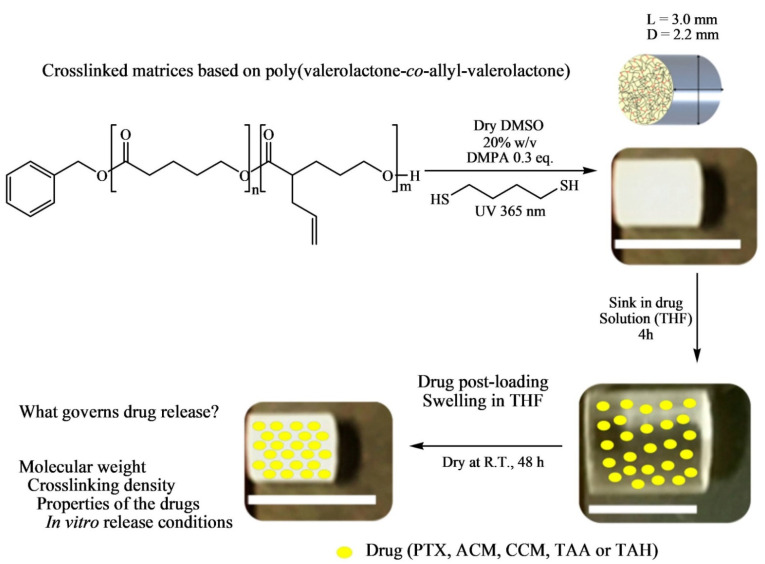
Schematic of the formation of the crosslinked polymer network and post-drug loading procedure in organic solvent of the IDDS as designed by Le Devedec et al. Adapted with permission from [60]; published by American Chemical Society, 2018.

The post-synthesis modification was performed via UV-mediated crosslinking using 1,6-hexanedithiol (HDT) as crosslinker to generate solid cylindrical amorphous or semicrystalline polymeric matrices as potential implantable drug delivery systems (IDDS) (Figure 7). In the post-functionalization, PVL-*co*-PAVL copolymers (0.25 mol equivalents of 2,2-dimethoxy-2-phenylacetophenone (DMPA)), and HDT crosslinker (0.5 functional group molar equivalents) were fully dissolved in dry dimethyl sulfoxide (DMSO) solvent under heating. A 1 mL syringe (inner diameter, i.d. = 4.7 mm and d = 5 cm) was used as a scaffold to inject in the solution, and then the syringe was UV-irradiated (λ 365 nm) for 20 min placed in an upright position. The crosslinked polymer (CP) obtained by this thiol-ene click chemistry was syringed out and purified by solvent exchange in THF (washed extensively) and dried at room temperature. The maximum content of AVL reported in the copolymer was 28%.

The conventional way of drug-loaded polymeric microparticle (MP) preparation consists of co-dissolving polymer and drugs in the organic phase, followed by oil-in-water emulsification. However, as the crosslinking reagent HDT and catalyst DMPA, need to be removed after post-particle formation, the conventional way is considered as an inappropriate technique for the preparation of drug-loaded crosslinked copolymer MPs. To mitigate this concern, the authors adopted the “post-loading” swelling-equilibrium method to load all drugs investigated in this study (including paclitaxel, triamcinolone acetonide and hexacetonide, curcumin, and acetaminophen) within the crosslinked PVL-*co*-PAVL MPs. For this purpose, swelling/equilibration of dried CPs in saturated drug solutions was formulated by equilibrating around 15 mg of CPs in a 30 mg/mL drug containing 0.5 mL of THF for 4 h, and subsequently removing the surface-adsorbed drug through a brief rinse in fresh THF for 10 s. Finally, the drug-loaded CPs were dried at room temperature to evaporate the THF solvent.

The authors observed the swelling behavior of the four crosslinked copolymer matrices (CPs), i.e., CP_15K_ (coral-like, heterogeneous surface morphology with densely packed folds), CP_32K_ (smooth surface with uniform ridges) CP_39K_ (smooth surface with uniform ridges), and CP_7.5K_ (smooth surface with uniform ridges), in the CH_2_Cl_2_, THF, toluene, DMSO, and H_2_O solvents. The degree of swelling was predicted by using the group contribution method (GCM), i.e., CH_2_Cl_2_ > THF > toluene > DMSO > H_2_O. The same trend was witnessed for all four copolymer systems, from CP_7_._5K_ to CP_39K_. Though, CP_39K_ and CP_32K_ implants swelled well and kept structural integrity in CH_2_Cl_2_, owing to the high molecular weight of copolymers, CP_7_._5K_ and CP_15K_ matrices broke into pieces after 2 h, accounted for by the low molecular weight of these copolymers. THF was chosen as the solvent for drug loading owing to the high degree of implant swelling without compromising structural integrity and drug solubility in THF. The four copolymer matrices swelled more in diameter than in length following 4 h of equilibration in THF.

From the same research group, Bao et al. [74] prepared PVL-*co*-PAVL MPs via an oil-in-water emulsification method, using PVA as stabilizer, followed by a thiol-ene click reaction, i.e., UV-assisted crosslinking of HDT reactive monomer. The objective was to introduce thiol functionality into the copolymer MPs and tailor the diameter of the MPs. In this formulation method, the authors prepared the oil phase by dissolving PVL-*co*-PAVL copolymer, DMPA photoinitiator, and HDT crosslinker in dichloromethane (DCM). The water phase consisted of 5 mL of deionized (DI) water containing 5% PVA (*w*/*v*). The oil phase was then incorporated into the water phase and homogenized with the help of a Polytron™ 2500E Homogenizer to produce a MP suspension. Next, the thiol-ene click reaction was conducted by UV-irradiating the MP suspension to assist crosslinking of the copolymer. After evaporating the solvent, the sample was filtrated using a cell strainer (Fisherbrand, 40 mm mesh) to retain MPs above the size cut-off. The filtration residue was then suspended in DI water, followed by centrifugation to remove the surfactant. After that, MPs were washed with acetone extensively to get rid of unreacted HDT and DMPA. The scanning electron microscopy (SEM) analysis displayed that MPs had a smooth spherical morphology with an average diameter of 66 ± 13 µm and the differential scanning calorimetry (DSC) and thermogravimetric analysis (TGA) analyses ensured that the crosslinking of the copolymer improved the integrity and thermal stability of the MPs.

In vitro evaluation of the Le Devedec study [60] showed that the PVL-*co*-PAVL MPs demonstrate sustained release of paclitaxel (PTX) for up to 19 days following first-order release kinetics where 60% cumulative fractional release was observed after six days, while 90% release was seen at the 19th day. In vitro degradation study indicated a slow but noticeable erosion, and thus suggested the applicability of these MPs for sustained drug release. The in vivo release of PTX from the MPs was found to be lower than predicted by the authors; based on the in vitro release studies, minimal tissue damage was observed at the administration site. The authors explained the lower release in the in vivo study to be due to the differences between the environment within the subcutaneous tissue (e.g., reduced aqueous volume) and in vitro release media.

**Figure 8:**
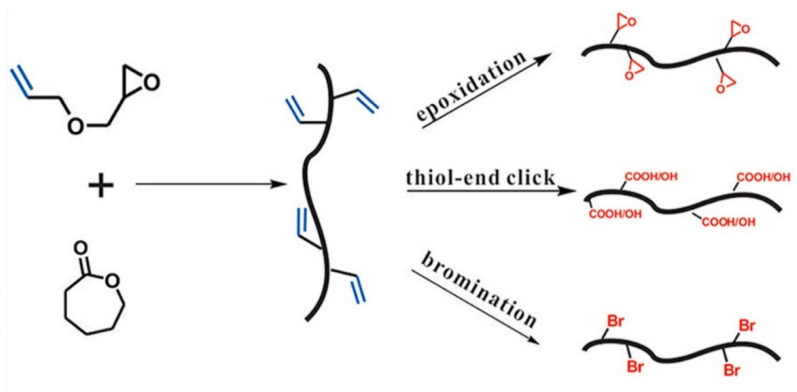
Schematic representation of pendant allyl groups containing poly(CL-AGE) copolymer synthesis and post-functionalization as reported by Yang et al. Reprinted with permission from [40]; Published by Elsevier, 2017.

Yang et al. [40] reported a scalable and facile strategy for the synthesis of an allyl functional aliphatic polyester via the ring-opening copolymerization of ε-caprolactone (CL) and AGE. This study also observed a similar trend report by Pound-Lana [36], i.e., an increment of epoxide monomer in the polymerization feed ratio (with maximum incorporation of 16.7%), leading to a higher number of reactive allylic groups in the copolymer. This study also investigated the effects of temperature on polymerization and found that the amount of AGE in copolymer and AGE homopolymer synthesis increases with increased temperature. To prepare new functionalized polyesters, the pendant allyl groups of poly(CL-AGE) copolymer were post-functionalized by thiol-ene click, epoxidation, and bromination reactions. For this purpose, 2-mercaptoethanol, 3-chloroperoxybenzoic acid, and bromine were employed to insert functionalities separately (Figure 8).

**Figure 9:**
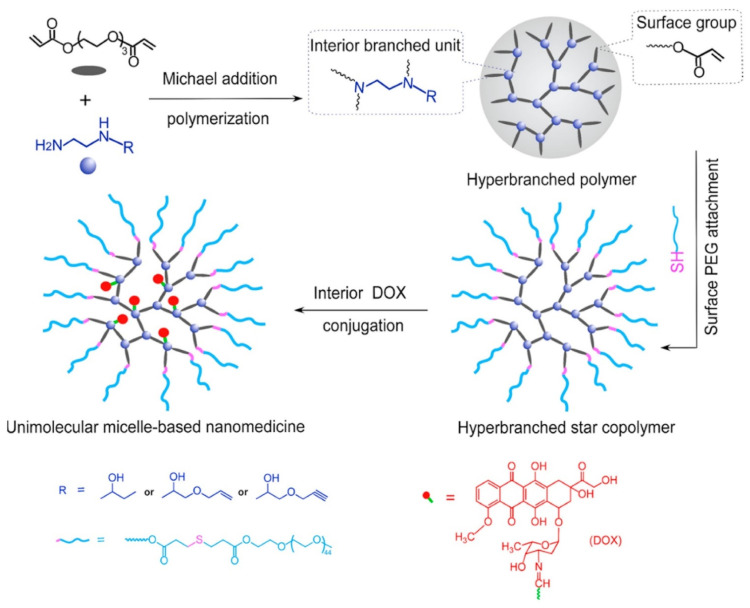
The synthesis of unimolecular micelle-based nanomedicines as described by Liang et al. Reprinted with permission from [75]; published by Elsevier, 2020.

To avoid the thermodynamic stability issue related to traditional polymeric micelles, Liang et al. [75] successfully designed and synthesized dense hyperbranched polymers with both terminal and internal “allyl” reactive groups. These reactive groups were subsequently utilized to attach hydrophilic chains at surface and interior drug conjugation (Figure 9). Firstly, the authors synthesized a series of new B′B_2_-R monomers, in which B stands for the hydrogen in primary amine, Bʹ stands for the hydrogen in secondary amine, while R is either the hydroxyl, alkene, or alkyne group that will not participate in the polymerization but will allow post-polymerization modification, by the ring-opening reaction of N-ethylethylenediamine with 1,2-epoxybutane, AGE, and propargyl glycidyl ether, called Hyperbranched poly(amino ester)s (HBPAE). Later, Poly(ethylene glycol) diacrylate (PEGDA), labelled as A_2_ monomer, was allowed to react with BʹB_2_-R monomers via Michael addition polymerization. The authors relied on the higher reactivity of primary amine in BʹB2-R to selectively react A_2_ monomer acrylate group to synthesize AB_2_ type intermediate with no gelation. Thereafter, hyperbranched star copolymers were synthesized via Michael addition reaction between PEG-SH, with terminal acrylate group of A_2_ preserving the internal R group, thus, attaching PEG chains onto the surface of HBPAE-R. DOX was linked to the internal R groups via an acid-labile hydrazide bond to achieve a pH-responsive drug release. The DOX loading content for PEG-HBPAE-DOX was calculated to be ~32.0 wt% through the ^1^H NMR analysis. The size obtained via transmission electron microscopy (TEM) for PEG-HBPAE-OH and PEG-HBPAE-DOX micelles was 15.2 ± 2.1 and 14.2 ± 2.8 nm, respectively, whereas the hydrodynamic diameter measured by dynamic light scattering (DLS) was 32.4 and 30.4 nm, respectively. The in vitro release study for PEG-HBPAE-DOX was conducted at pHs of 7.4 and 6.0 to mimic normal extracellular pH and tumor microenvironment pH, respectively. The release rate observed at pH = 6.0 was higher (up to 95% after 48 h) compared to release at pH = 7.4, which reached a plateau after 6 h, indicating high stability under physiological conditions and pH-dependent drug release. The in vitro cytotoxicity of DOX, PEG-HBPAE-OH, and PEG-HBPAE-DOX was assessed on HeLa cells where the free nanocarrier exhibited almost no cytotoxicity even at high concentration (1.0 μg mL^−1^), while the cell viability was only 16.0% in the PEG-HBPAE-DOX samples, with a drug concentration of 1.0 μg mL^−1^.

**Figure 10:**
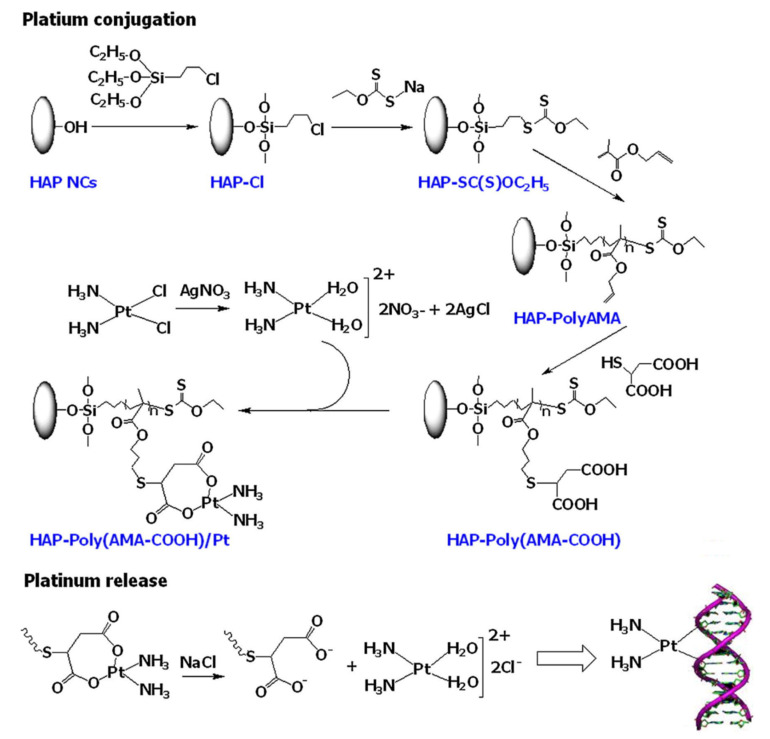
Synthetic route for HAP-PolyAMA nanohybrids via RAFT polymerization and cisplatin conjugation with a possible drug release mechanism as designed by Bach et al. Reprinted with permission from [76]; published by Elsevier, 2013.

Bach et al. [76] reported a facile method to synthesize a polymer grafted hydroxyapatite (HAP)-based drug delivery system for controlled and targeted drug release. Unfortunately, HAP is not degradable in the human body, and therefore introduction of polymer moieties to the backbone of HAP has been found to be a good strategy to improve the physicochemical properties of HAP. In this study, they utilized poly(allyl methacrylate) (Poly-AMA) to initially encapsulate HAP nanocrystals (NCs) through surface-initiated RAFT (SI-RAFT) polymerization. Thereafter, the pendant alkene in PolyAMA polymers was utilized to introduce carboxylic functionality via a thiol-ene reaction. Subsequently, PEG-HBPAE-DOX the HAP-Poly(AMA-COOH)/Pt complex was prepared by the interaction of HAP-PolyAMA nanohybrids with transiently generated cis-diamminediaqua platinum (II) species through the hydrolysis of cisplatin (Figure 10). Field emission scanning electron microscopy (FE-SEM) revealed the morphology of HAP-Poly(AMA-COOH)/Pt complex, with Pt particles in the range of 10–50 nm decorating the surface of the HAP-Poly(AMA-COOH) nanohybrids. The release of the Pt species from the HAP-Poly(AMACOOH)/Pt complex in phosphate-buffered solution (pH = 7.4) and acetate buffer solution (pH = 5.0) was found to be pH-dependent. A faster release was observed in acidic pH, which is attributed to the hydrolysis of the HAP-Poly(AMA-COOH)/Pt complex.

The toxicity levels of HAP-Poly(AMA-COOH)/Pt complex and pure cisplatin were assessed against HeLa cells and A549 cells for 24, 48 h, and 72 h using MTT assay. Cisplatin and HAP-Poly(AMA-COOH)/Pt complex showed a dose-dependent cytotoxicity and enhanced cytotoxicity with longer incubation times. The cytotoxicity of pure cisplatin was higher compared to the HAP-Poly(AMA-COOH)/Pt complex against A549 cells at 24 h, and the cytotoxicity became more prominent when incubated for 48 h. Pure cisplatin also showed a higher cytotoxicity at 24 h on HeLa cells compared to the HAP-Poly(AMA-COOH)/Pt complex; however, the effect became nearly identical on prolonged exposure. Hence, HAP-Poly(AMA-COOH)/Pt complex nanohybrid is a promising nanocarrier for controlled drug delivery.

**Figure 11:**
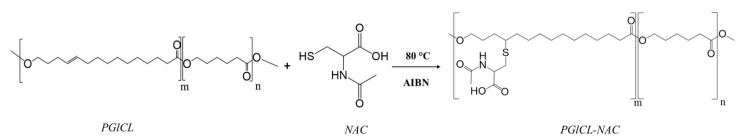
The functionalization of Poly(globalide-*co*-ε-caprolactone) side-chain with N-acetylcysteine via a thiol-ene reaction as depicted by Guindani et al. Reprinted with permission from [77]; published by Elsevier, 2019.

Polyglobalide (PGl) is a biocompatible and non-toxic polyester that is obtained through ROP of globalide and retains the double bond on its main skeleton after polymerization. Thus, the design of copolymers of CL and globalide (Gl) would add wide versatility to the polymer’s biomedical applications as it opens doors for post-polymerization modification via thiol-ene coupling reaction. Therefore, Guindani et al., [77] incorporated the antimucolytic and antioxidant, N-acetylcysteine (NAC), to poly(globalide-*co*-ε-caprolactone) (PGlCL) copolymer. NAC is a hydrophilic molecule and bears a thiol group that allows conjugation by thiol-ene reaction; moreover, the formed thio-ether linkages could enhance the affinity of the polymer for water and reduce its degree of crystallinity. Herein, PGlCl was synthesized by ROP using supercritical carbon dioxide (scCO_2_) as solvent in a fixed mass ratio of 1:2 (CO_2_:monomers) with variation of the Gl/CL mass ratios as follows: 10/90, 25/75, 50/50, 75/25, and 90/10. Thereafter, PGlCL with varying Gl/CL ratios were reacted with NAC via thiol-ene reaction resulting in the functionalized copolymer, PGlCL-NAC (Figure 11). The functionalization of PGlCL containing a 10/90 Gl/CL resulted in lower Tm PGlCL-NAC samples; however, the degree of crystallinity (χ_c_) did not practically change after functionalization. It was suggested that this result could be ascribed to weaker intermolecular forces in the crystalline arrangement of the material due to the addition of NAC. The free volume increase due to branching, which allows for an easier chain movement of the chains, reduces the energy needed to overcome the secondary intermolecular forces between the chains of the crystalline phase. Generally, amorphous polymers exhibit higher degradation rates than semi-crystalline polymers, and hence they are promising candidates for biomedical applications. Polymer samples with amorphous characteristics were then assessed for their water affinity through contact angle assay. PGlCL with different Gl/CL ratios presented contact angle values around 88°, and accordingly may be considered hydrophobic materials. However, PGlCL-NAC demonstrated lower contact angle values, varying from ~60° to ~47°. The contact angle decrease as a result of the increased hydrophilicity via the attachment of the hydrophilic NAC yields a polymer more suitable for cell attachment and drug delivery, and allows for a better degradation rate. NAC is a renowned antioxidant and, consequently, after functionalization, PGlCL-NAC also presented antioxidant activity. PGlCL-NAC with a Gl/CL ratio of 50/50 was assessed for its antioxidant potential through 1,1-diphenyl-2-picrylhydrazil (DPPH) and 2,2′-azino-bis(3-ethylbenzothiazoline-6-sulphonic acid) (ABTS) assays. The non-functionalized PGlCL showed no antioxidant activity in both assays while free form of NAC showed a strong antioxidant activity in DPPH (EC50 = 4.31 ± 0.03 μg mL^−1^) and ABTS (EC50 = 137 ± 3 μg mL^−1^). PGlCL-NAC presented EC50 = 4065 ± 157 μg mL^−1^ and EC50 = 1553 ± 22 μg mL^−1^ in DPPH and ABTS assays, respectively.

### 2.2. Polyethers

Insertion of allyl functional (poly)glycidyl ether as building blocks in polyether- leads to a wide range of promising applications for these materials. In this section, we have discussed the synthesis of such polymers and its post-functionalization, if any.

**Figure 12:**
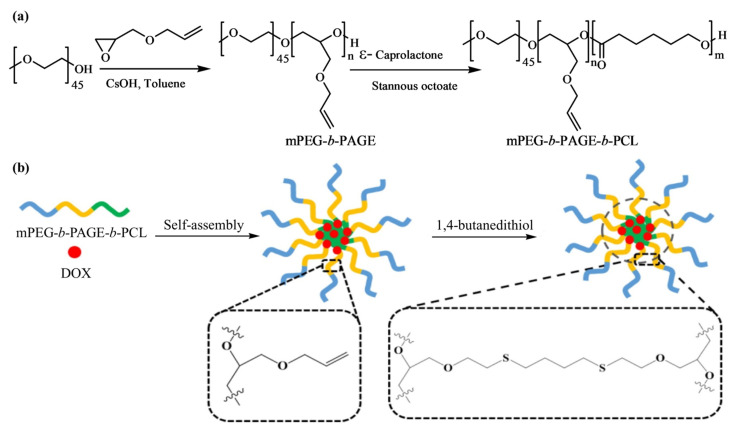
The synthetic procedure of mPEG-*b*-PAGE-*b*-PCL (**a**) and the preparation of the DOX-loaded CLM by “thiol-ene” reaction (**b**) as described by Lu et al. Adapted with permission from [51]; published by Elsevier, 2020.

Lu et al. [51] designed and synthesized amphiphilic triblock copolymer, methoxy poly(ethylene glycol)-*b*-poly(allyl glycidyl ether)-*b*-poly(ε-caprolactone), (mPEG-*b*-PAGE-*b*-PCL) with different hydrophobic lengths using two successive ROPs and post-functionalized by a thiol-ene click reaction, displayed in Figure 12. The macroinitiator mPEG−OH was employed for the deprotonation of the terminal -OH group and then subjected to the anionic ROP of AGE to obtain mPEG-*b*-PAGE diblock copolymers. Later, different amounts of CL were incorporated into the system to synthesize the triblock copolymer mPEG-*b*-PAGE-*b*-PCL with distinct hydrophobic lengths.

Blank micelles were prepared by dissolving mPEG-*b*-PAGE-*b*-PCL triblock copolymers in THF, followed by adding copolymer solution into pure water drop-wise at room temperature with continuous stirring. The blank micelles were obtained by evaporating THF solvent through rotary evaporation. To prepare crosslinked mPEG-*b*-PAGE-*b*-PCL triblock copolymers micelles, 1,4-butanedithiol crosslinker was added into the micelle dispersion solution and irradiated the dispersion by a UV lamp (λ 365 nm). The centrifugal ultrafiltration technique was applied to remove residual crosslinker and cut off low-molecular weight fraction (M.W. 3500).

The triblock copolymers could self-assemble into highly stable polymeric spherical micelles in an aqueous solution. The micelles formation depends on the hydrophobic interactions between the drug and the hydrophobic segments of the amphiphilic polymers. The micelles encapsulate DOX and subsequently undergo interface crosslinking by a thio-ene reaction with 1,4-butanedithiol. The covalently crosslinked network ultimately enhances the stability of the micelles but decreases its size to some extent, compared to that of the non-crosslinked micelles. The authors attributed this slight decrease in size to the shrinkage of the inner core of the micelles. The DLS results indicate that the diameter of the DOX-loaded micelles was dependent on the hydrophobic PCL block length in the copolymer composition. The maximum drug loading content and entrap efficiency were calculated as 8.62% and 47.16%, respectively, for the mPEG-*b*-PAGE-*b*-PCL_50_/DOX micelles with a size of 99.28 nm. The in vitro cytotoxicity was determined by MTT assay with human oral epidermoid carcinoma (KB) and human gastric carcinoma (SGC) cell lines. The results showed that the DOX-loaded micelles could be effectively endocytosed by cancer cells and possessed good antitumor efficacy. In addition, pH-responsive DOX release profile was observed for both DOX-loaded noncrosslinked micelles (NCLMs) and crosslinked micelles (CLMs). As DOX is known for its enhanced protonation affinity and higher solubility in acidic environments, micelles achieved a rapid drug release in a weakly acidic intracellular environment. However, the cumulative release rate of DOX-loaded CLMs was lower than that of the DOX-loaded NCLMs at both pH 7.4 and 5.0 because the crosslinking layer hindered the release of DOX from the core of the micelles. The authors also investigated the tumor targeting efficiency of CLMs and NCLMs using in vivo fluorescence imaging in 4T1 tumor-bearing BALB/c mice; the dye used was the commercially available lipophilic dye DiR. They observed that the CLMs rapidly accumulated in tumors and had a better passive targeting ability than NCLMs due to their good stability, as the fluorescence intensity of DiR-loaded CLMs in the tumor (13.3%) was 1.67-fold higher than that of the NCLMs (9.3%). The authors summarized that the interface crosslinking strategy from the triblock copolymer mPEG-*b*-PAGE-*b*-PCL could improve the stability of the micelles in vivo and holds great promising applications in future cancer therapy.

**Figure 13:**
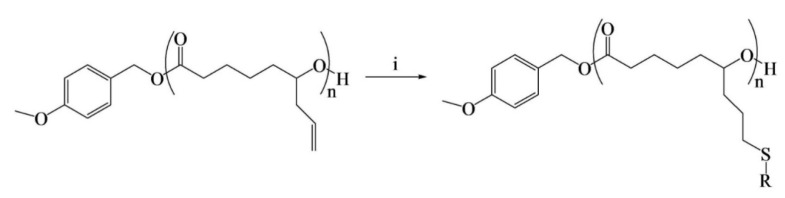
Synthesis of thiol-functionalized poly(ε-allyl-ε-caprolactone). Conditions: (i) IRGACURE 819, alkyl-thiol, chloroform, UV light (315–400 nm), RT as designed by Clamor et al. [78].

Clamor et al. [78] reported a well-controlled ROP of ε-allyl caprolactone using Mg(BHT)_2_(THF)_2_ to yield an allyl-functionalized lactone, ε-allyl-ε-caprolactone (AεPCL), suitable for post-polymerization modification with a 95% monomer conversion (Figure 13). The synthesized PCL was subsequently post-functionalized by photo-initiated thiol-ene addition on the pendant allyl-functionality using various alkyl thiols to produce lipophilic polyesters with tuned lipophilicity and crystallinity. An increasing solubility in n-dodecane was observed with increased alkyl chain length on the PCL backbone. Furthermore, crystallinity increased with alkyl chain length; a change in crystallinity from amorphous to semicrystalline was observed when the alkyl length reached 10 carbon atoms in length, likely due to effective chain interaction among the alkyl pendant groups. The authors demonstrated that the physical and thermal properties of PCL can be altered by varying the alkyl functionality. The tailoring of the polymer microstructure and solubility could be advantageous in exploring PCL in new nanostructures and (bio)material applications.

**Figure 14:**
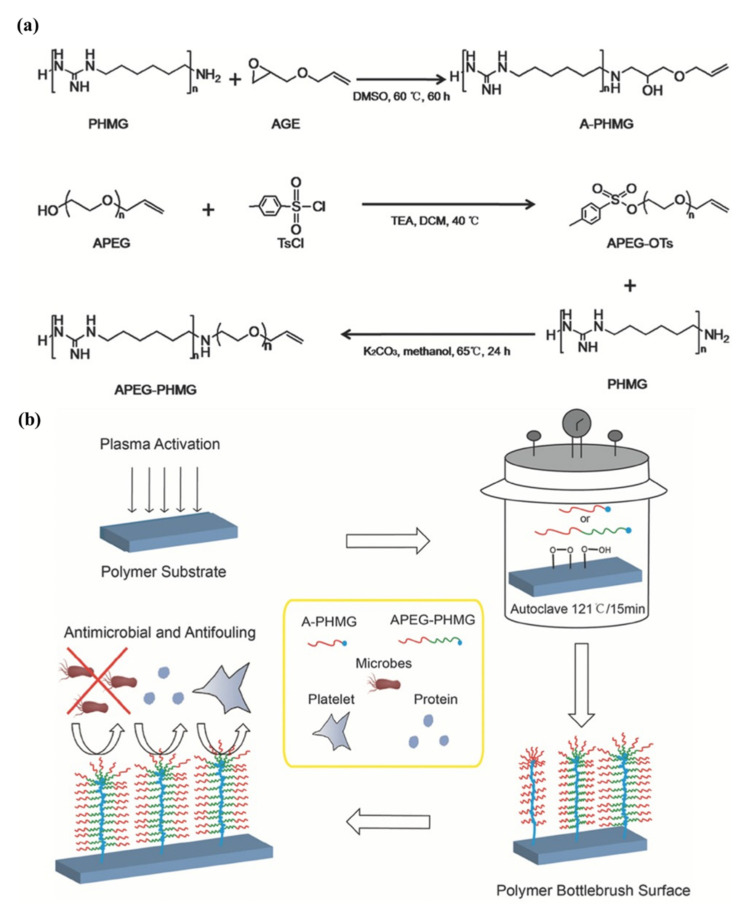
(**a**) Synthesis of A-PHMG and APEG-PHMG oligomers. (**b**) Schematic of plasma/autoclave-assisted grafting of A-PHMG/APEG-PHMG bottlebrushes on polymeric substrates as studied by Su et al. Reprinted with permission from [37]; published by Wiley, 2017.

Su et al. [37] successfully designed and synthesized allyl-terminated polyethylene glycol-polyhexamethylene guanidine block copolymer, APEG-PHMG, consisting of antimicrobial, antifouling, and surface-tethering segments in a facile, up-scalable, and inexpensive way. Later, APEG-PHMG oligomers were grafted on polydimethylsiloxane (PDMS) silicone rubber substrates by a facile plasma/autoclave-induced surface grafting polymerization method to form a permanently bonded bottlebrush-like surface coating. The authors basically modified the –NH_2_ end-group of polyhexamethylene guanidine (PHMG) with AGE and, ultimately, obtained allyl-functionalized oligomers. The authors first prepared hetero-bifunctional PEG with allyl and tosyl groups (APEG-OTs) using PEGs with 1200 and 2400 Da molecular weights, where end hydroxyl group of APEG was reacted with 4-toluene sulfonyl chloride (TsCl) to produce APEG-OTs. After that, thermally and chemically stable broad-spectrum antimicrobial agent polyhexamethylene guanidine (PHMG) was conjugated with APEG-OTs to produce the block copolymer (APEG-PHMG). Separately, an allyl-terminated PHMG was synthesized by reaction with AGE. The synthesis process of oligomers and schematic of plasma/autoclave-assisted grafting is depicted in Figure 14. Argon plasma (13.56 MHz, 40 W, and 25 sccm) was applied on the PDMS surface for 5 min to generate reactive free radicals and, subsequently, plasma-treated PDMS was exposed to air for 15 min to produce relatively stable peroxide or hydroperoxide groups on the treated surface via reaction between reactive free radicals on the PDMS surface, and oxygen and water in the air. The activated PDMS surfaces were then immersed in 5 wt% of A-PHMG or APEG-PHMG oligomer solution in vials, separately. After that, sample vials were autoclaved at 121 °C for 15 min. In this autoclaving process, reactive free radicals were generated again on the PDMS surface through decomposition of peroxide/hydroperoxide groups on the silicone surface under high autoclaving temperature. These newly generated free radicals initiated the polymerization of the allyl groups. The main function of allyl functionality of A-PHMG/APEG-PHMG oligomers was the formation of a permanent covalent bond between oligomers and the plasma-activated silicone surfaces, using allyl group as this group is very reactive to peroxide/hydroperoxide groups generated on silicone substrate via plasma activation and the reaction is very fast.

The antimicrobial activity of AGE and APEG modified PHMG oligomers were examined both in solution and coating form against gram-negative, gram-positive bacteria, and fungus. In this study, Pseudomonas aeruginosa (ATCC 27853), Staphylococcus aureus (ATCC 2921), and *Fusarium solani* (ATCC 36031) were considered as gram-negative, gram-positive bacteria, and fungus, correspondingly. In solution form, a broth microdilution minimum inhibitory concentration (MIC) assay was performed to determine the antimicrobial activity; results are shown in Table 2. The authors observed that both A-PHMG and APEG1_200_/_2400_-PHMG coatings displayed potent broad-spectrum and reusable antimicrobial activity against gram-positive bacterium (*S. aureus*), gram-negative bacterium (*P. aeruginosa*), and fungus (*F. solani*), whereas APEG_1200_/_2400_-PHMG coatings exhibited superior antifouling activity and long-term reusability to A-PHMG coating. Contrarily, APEG_2400_-PHMG coating demonstrates the most effective in vitro anti-biofilm and protein/platelet-resistant properties, as well as excellent hemo/biocompatibility. In addition, APEG_2400_-PHMG showed high efficacy towards the inhabitation of the bacteria growth, significantly, and prevented implant-associated infection caused by *P. aeruginosa* in a rodent subcutaneous infection model.

The outcomes of the study indicated that antimicrobial and antifouling APEG-PHMG dual-functional diblock copolymer coating has great potential to prevent bacterial colonization and biofilm formation on biomedical implants, and subsequently in combating biomedical devices/implant-associated infections.

### 2.3. Poly(Ester-Anhydride)s

Poly(ester-anhydride) copolymers are frequently synthesized to combine the individual properties of polyester and polyanhydride polymers in a single polymer, with various applications. Polyesters display mostly bulk erosion mechanisms and polyanhydrides exhibit surface erosion mechanisms during hydrolytic degradation, whereas poly(ester-anhydride)s demonstrate a two-stage degradation mechanism. In poly(ester-anhydride)s, first, a rapid degradation in molecular weight of the polymer occurred through polymer chain breaks at the hydrolytically labile anhydride linkage, and then, a slower degradation of remaining oligomers took place. The rate of the second stage, the degradation of the remaining oligomers, is determined by the composition of polyester prepolymers [79]. Numerous studies have been conducted to prepare functional poly(ester-anhydride)s bearing allyl pendant groups to combine the individual properties of polyesters and polyanhydrides, and to control the degradation rate and mode of degradation by manipulating the polymer composition. 

Jaszcz et al. published three separate studies discussing the synthesis and post-functionalization of functional poly(ester-anhydride)s, based on succinic acid [48], on oligosuccinate, and aliphatic diacids. Furthermore, they studied the hydrolytic degradation behavior [49] and epoxidation of pendant allyl groups in poly(ester-anhydride)s, proposed for application in drug delivery [50]. The purpose of introducing allyl groups to poly(ester-anhydride)s was that the allyl groups could be converted into epoxide groups through epoxidation reaction, and the epoxide functionality in poly(ester-anhydride)s could create an interesting perspective for chemical coupling of drugs to poly(ester-anhydride)s carrier, e.g., via microspheres.

**Figure 15:**
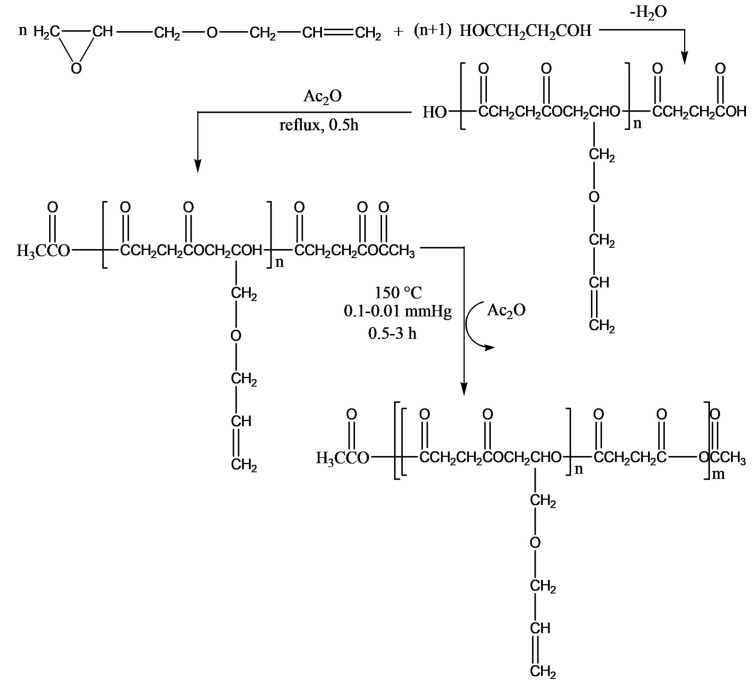
Polycondensation of succinic acid and allyl glycidyl ether, conversion of carboxyl end groups of the macromer to anhydride groups, and preparation of poly(ester-anhydride)s by melt polycondensation. Adapted with permission from [48]; published by Elsevier, 2008.

The authors described a three-step synthesis of succinic acid-based functional poly(ester-anhydride)s bearing allyl groups in the side chains, as shown in Figure 15. The steps include (1) preparation of carboxyl-terminated functional oligoesters (OSAGE) by melt condensation of AGE with an excess of diacid (DA), succinic acid, or carbonic acid; (2) conversion of carboxyl end groups of the macromer to mixed anhydride groups by refluxing in acetic anhydride; and (3) preparation of poly(ester-anhydride)s from ester-anhydride prepolymers by melt polycondensation.

**Figure 16:**
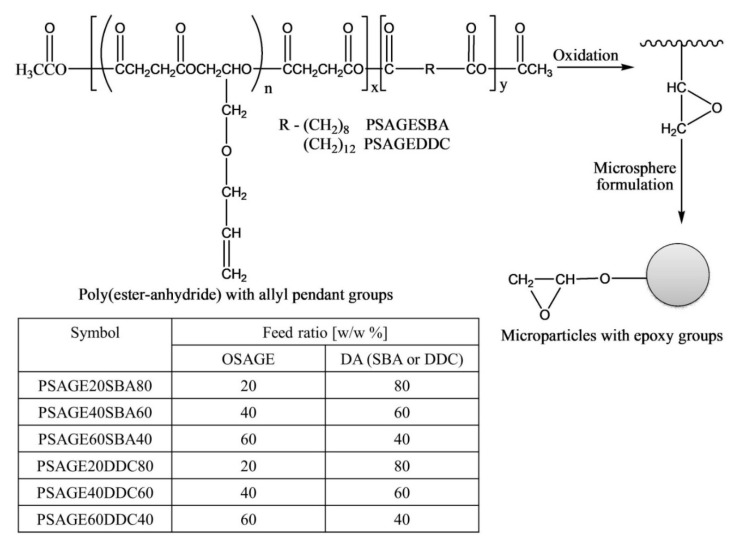
Preparation of microspheres with surface epoxy groups as described by Jaszcz et al. Adapted with permission from [50]; published by Elsevier, 2012.

The authors post-functionalized poly(ester-anhydride) with allyl groups to epoxy groups via oxidation using m-chloroperbenzoic acid (MCPBA) with 100% conversion (Figure 16) [50]. However, it was observed that the conversion of allyl groups into glycidyl was greatly dependent on the content of pendant allyl groups in poly(ester-anhydride) (OSAGE to DA ratio), the concentration of a polymer in the reaction solution, the amount of MCPBA, and the reaction time.

**Figure 17:**
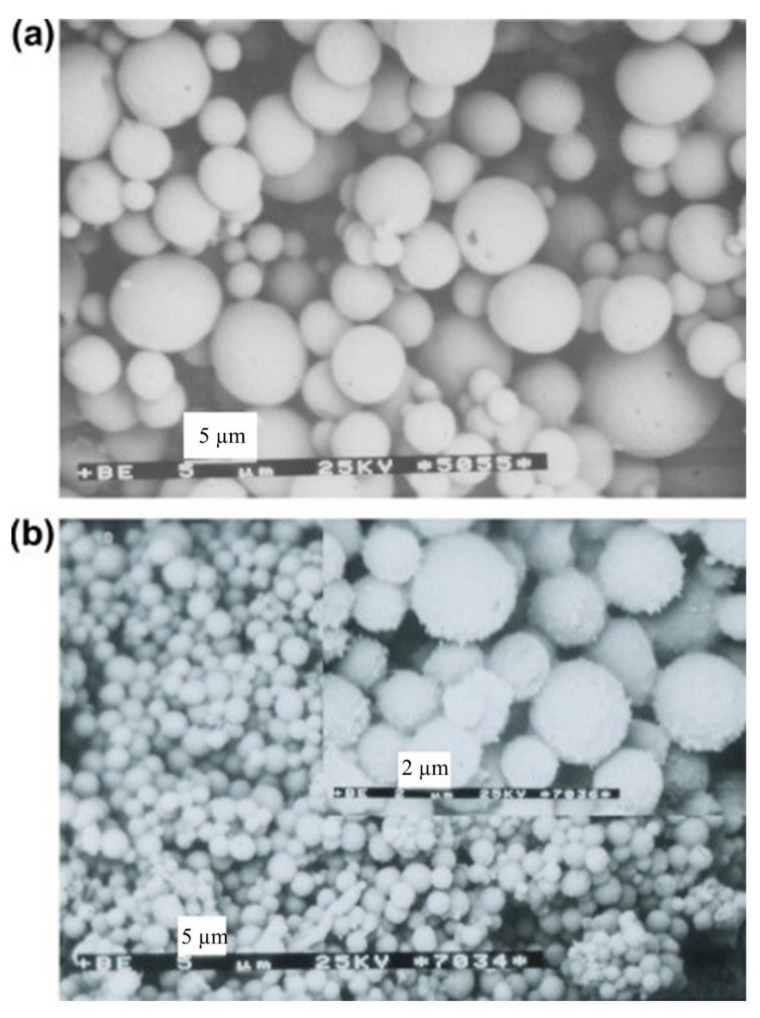
Representative SEM microphotographs of microspheres obtained from the initial (**a**) and oxidized (**b**) PSAGE20SBA80, as studied by Jaszcz et al. Adapted with permission from [50]; published by Elsevier, 2012.

The microspheres were fabricated by emulsion solvent evaporation method using parent and oxidized polymers. The microparticles obtained in this study were stable and spherical in shape (with diameter 4.7–9.2 μm in size distribution D_v_/D_n_ = 1.2–1.4) and no holes or pores were observed on their surfaces. Microspheres obtained from allyl functional polymers were smoother, bigger, and had a broader size distribution in comparison with microspheres prepared with epoxy-functional polymers (Figure 17). Authors also suggested that, owing to the higher polarity of epoxide groups with respect to allyl groups, the epoxy-functionalized microspheres did not show agglomerations and coalescence in an aqueous medium. Moreover, the epoxide groups can be covalently bonded with the functionalities of drugs, such as amines, and can be potentially utilized as polymeric drug carriers [50]. In the preliminary trials, the authors conducted several reactions between amine-containing model compounds, such as isopropylamine, a-amino-x-methoxy-poly(ethylene glycol)s, spermine, and spermidine, and the epoxy-functionalized poly(ester-anhydride) microspheres. It was observed that the model amine compounds can covalently bond to the surfaces of the microspheres, which was confirmed by the ^1^H NMR or ATR-IR spectra as epoxy groups peaks disappeared. However, the consumption of anhydride bonds was also observed simultaneously. The two factors which determined the ratio of epoxide groups to anhydride bonds consumption were the basic character of amine compounds and the reaction conditions.

Both initial and oxidized poly(ester-anhydride)s microspheres were subjected to hydrolytic degradation in phosphate buffer (pH = 7.4) at 37 °C for 10 days, followed by weight loss determination due to hydrolytic degradation as a function of immersion time in buffer solution by gel permeation chromatography (GPC). Two general trends were identified from GPC results: (1) among the microspheres prepared from unoxidized and oxidized poly(ester-anhydride)s, the unoxidized microspheres degraded somewhat faster than the respective oxidized ones, and (2) microspheres obtained from poly(ester-anhydride) containing sebacic acid (SBA) degraded faster compared to poly(ester-anhydride) containing dodecanedicarboxylic acid (DDC) microspheres. After 7 days of hydrolytic degradation, the weight loss was calculated. For the unoxidized and oxidized PSAGE20SBA80 microspheres, approximately 90% and 60% weight loss was observed, respectively. Whereas a weight loss of 54% and 45% was determined for unoxidized and oxidized PSAGE20DDC80 microspheres, respectively. Both oxidized SAGE20DDC80 and PSAGE20SBA80 microspheres produced water soluble products by hydrolytic degradation. However, oxidized SAGE20DDC80 microspheres took longer to degrade entirely (more than a month) in comparison to oxidized SAGE20DDC80 microspheres (over three weeks).

**Figure 18:**
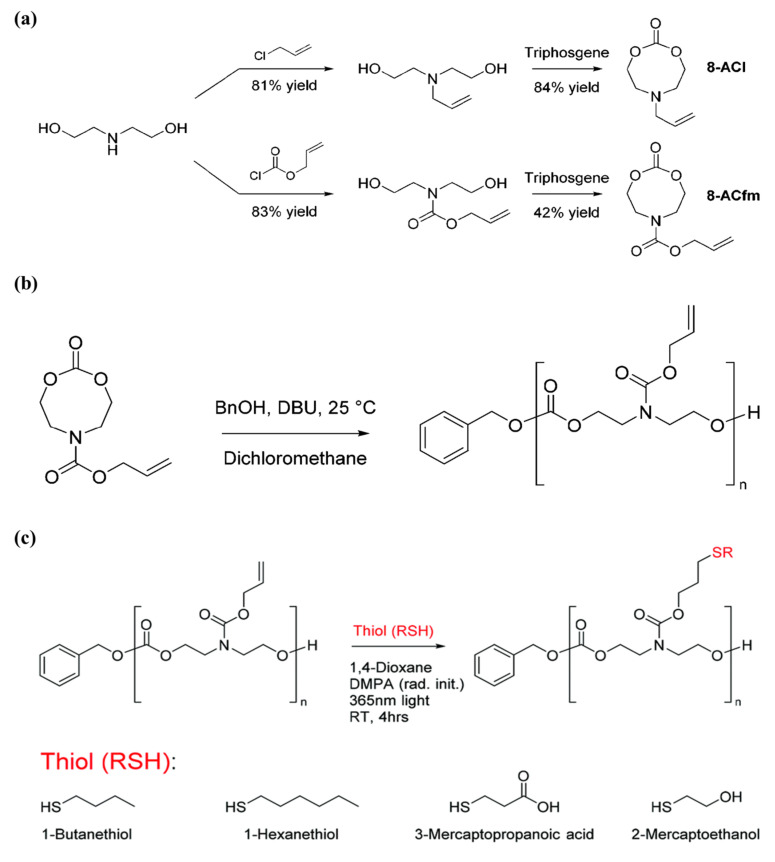
(**a**) Synthesis scheme for allyl bearing eight membered cyclic carbonates, (**b**) ring-opening homopolymerization of 8-ACfm, and (**c**) thiol-ene reaction on 8-ACfm, as proposed by Yuen et al. Adapted with permission from [80]; published by The Royal Society of Chemistry, 2018.

Aliphatic polycarbonates are degradable materials with low toxicity and high compatibility, and thus are an important class of polymers in biomedical applications. Designing and polymerizing cyclic carbonates carrying allyl pendants that could be easily modified with thiol–ene reaction is a dominant approach in post-polymerization functionalization for polymers based on cyclic carbonates. The method is selective, facile, and yields high conversions and high reaction rates; however, the long reaction time needed for the allyl functional monomer to yield high conversions while maintaining its end-group fidelity remains a challenge. Therefore, Yuen et al. [80] incorporated allyl functionalities into N-substituted eight-membered cyclic carbonates, renowned for being more reactive than six-membered cyclic carbonates, to improve the reactivity of cyclic carbonate monomers that contain allyl functionality. The authors further utilized the allyl groups in the polymer by enabling modification of the polymer and, hence, widening the extent of the versatility of the carbonate-based polymers. Firstly, the authors synthesized two different allyl-bearing N-substituted eight-membered cyclic carbonates, 6-allyl-1,3,6-dioxazocan-2-one (8-ACl) and allyl 2-oxo-1,3,6-dioxazocane-6-carboxylate (8-ACfm), in yields of 84% and 42%, respectively, as shown in Figure 18A. Thereafter, homopolymerization of the monomers 8-ACl and 8-ACfm was explored using the organocatalyst 1,8-di- azabicyclo[5 .4.0]undec-7-ene (DBU) (Figure 18B). They used 10% DBU for catalysis and monitored the polymerization and conversion through ^1^H NMR, in which 8-ACfm was able to achieve 97% monomer conversion in 10 min; on the other hand, 8-ACl reached only 14% monomer conversion after 1 h. The observed variability in reactivity between 8-ACl and 8-ACfm was attributed to the different reactivity of the N-substituent pendant group of the cyclic carbonate between the two monomers. Later, the allyl bearing polymer was then modified with four thiols, 1-butanethiol, 1-hexanethiol, 3-mercaptopropionic acid, and 2-mercaptoethanol, as presented in Figure 18C. The efficiency of the modifications was very high with ≥90% conversion for all cases, as calculated by ^1^H NMR spectroscopy. Moreover, the authors managed to successfully form a gel through reacting allyl-bearing polycarbonates, with HDT as a crosslinker. Importantly, the superior polymerization kinetics of 8-ACfm allows its copolymerization with other cyclic monomers in a reasonable amount of time. The N-substituted monomer, 8-ACfm, was copolymerized with the commercially available trimethylene carbonate (TMC) with ≥97% within 1 h. The thiol–ene radical additions with 1-butanethiol were performed successfully for the prepared co-polymer, which suggested that N-substituted eight-membered cyclic carbonates chemistry is an attractive approach for producing functional biodegradable aliphatic polycarbonates.

### 2.4. Polysaccharides

Polysaccharides derived from natural resources are renewable, inexpensive, often biodegradable, biocompatible, generally nontoxic, and demonstrate excellent properties, including aqueous solubility, stability, and excellent swelling ability. Owing to these outstanding characteristics, polysaccharides are being utilized as suitable biomaterials in many biomedical applications, such as the delivery of drugs and therapeutics, protein encapsulation, wound healing, tissue regeneration, and bioimaging. Among many widely-known polysaccharides, chitosan (CS), derived from chitin via de-acetylation, primarily composed of D-glucosamine and N-acetyl glucosamine units with a β-(1–4)-linkage, is the second most abundant natural polysaccharide, after cellulose. Its biodegradable, biocompatible, non-toxic, and non-allergenic properties, as well as its antimicrobial, antioxidant, anti-tumor, and anti-inflammatory activities, make chitosan one of the most studied and frequently investigated biomaterials for a wide range of biomedical applications. Owing to its important biological properties, chitosan is considered an immunoadjuvant, anti-thrombogenic, and anti-cholesteric agent [81,82,83,84]. It is a very popular excipient in the pharmaceutical industry. Several research innovations have been made on chitosan as a polymer matrix for drug delivery [81,85], tissue engineering, and regenerative medicines. On account of its high versatility, chitosan can be processed into many physical forms, such as micro or nano-sized particles, fibers, gels, beads, films, sponges, scaffolds etc., for oral and parenteral drug delivery, and tissue engineering [86,87]. End group functionalization of chitosan by inserting allyl (ene) functionality opens new doors for promising applications of these materials. In this section, we have discussed the synthesis of such allyl-functionalized chitosan-based polymers.

**Figure 19:**
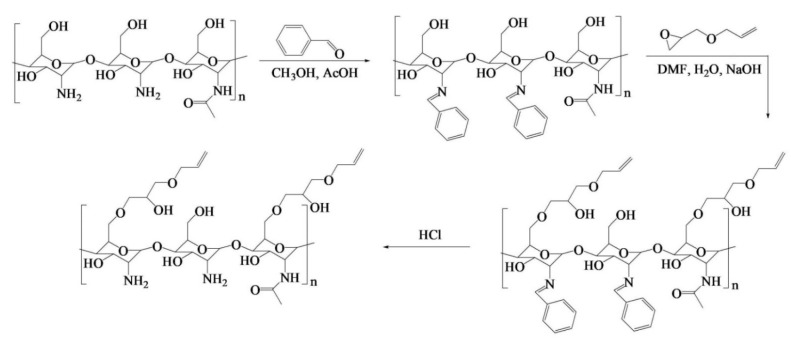
Synthesis route of OAL-CS as designed by Ding et al. Adapted with permission from [88]; published by Elsevier, 2020.

**Figure 20:**
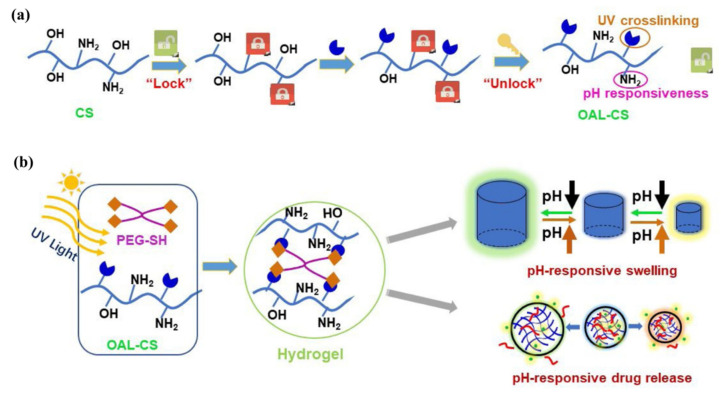
Schematic synthesis of (**a**) pH-responsive UV-crosslinkable C_6_ O-allyl chitosan (OAL-CS) via protection/deprotection of amino groups and (**b**) the OAL-CS hydrogel via UV-triggered “thiol-ene” click chemistry in which 4-arm PEG-SH served as crosslinker, as designed by Ding et al. Reprinted with permission from [88]; published by Elsevier, 2020.

Ding et al. [88] engineered a pH-responsive UV-crosslinkable C_6_ O-allyl chitosan (OAL-CS) polymer hydrogel. The authors synthesized OAL-CS via a three-step reaction: (1) a Schiff’s base reaction of C_2_-NH_2_ with benzaldehyde to suppress the activity of the amino group, (2) a ring-opening reaction of epoxy group of AGE with C_6_-OH of CS to graft UV-crosslinkable allyl groups on CS, and (3) removal of protective groups in dilute hydrochloric acid, as depicted in Figure 19. The authors adopted the protection of amino groups to preserve the pH responsiveness of the native CS and to ensure that the epoxy groups reacted solely with the hydroxyl groups (C_6_-OH) (Figure 20a). The authors post-functionalized OAL-CS via UV-induced “thiol-ene” click chemistry to demonstrate the drug delivery capability of the polymer (Figure 20b).

Hydrogel of OAL-CS was prepared by dissolving the polymer in PBS containing four-arm PEG-SH as crosslinker. The final mixture solution was turned into gel within 30 *s* under low-dose UV irradiation. Later, the pH-responsive swelling and shrinkage for modulating the small molecular drug, DOX, and macromolecular drug, bovine serum albumin (BSA) release, were investigated in PBS buffer solutions at pH = 5.0, 6.8, and 8.0. The authors found that the release behavior of the two drugs was different, and significantly dependent on the pH of the solution, while the release of the drugs occurred mainly by their free diffusion from the hydrogel. In the case of DOX, about 96% of the DOX load was released within 4 h in basic pH (pH = 8.0 PBS) and this value was 53% more than the DOX released within the same duration in neutral pH (pH = 6.8 PBS). On the other hand, after three days, the cumulative release of BSA at pH = 8.0 was only 27%, while 53% and 62% releases were observed at pH = 6.8 and 5.0, respectively. The pH-responsive shrinkage behavior of hydrogel delayed the release of BSA by 49%, whereas the free diffusion, together with extrusion of hydrogel, surprisingly promoted the release of DOX by 81%. It is noted that, due to the degradation of hydrogel in acidic pH = 5.0, a quick release of BSA was observed after 6 days. The product developed in this study has the properties of being worked as a patterned microgel and rapid transdermal curing hydrogel in vivo, with potential for pH-responsive drug delivery and other biomedical applications.

**Figure 21:**
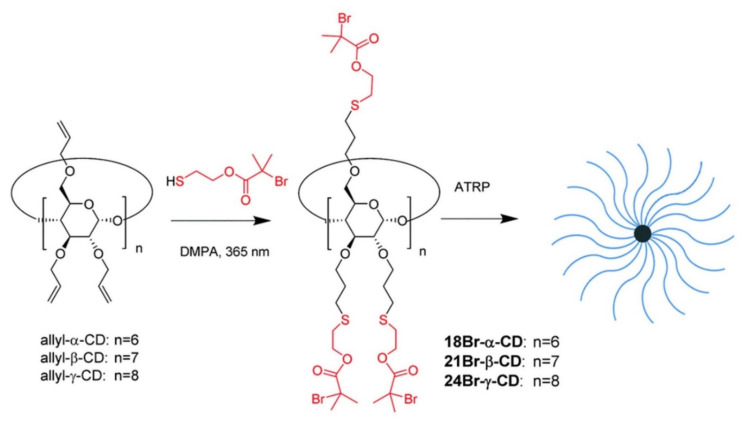
The synthetic route of multiarm star polymers from fully functionalized CD cores, as proposed by Yi. Reprinted with permission from [89]; published by The Royal Society of Chemistry, 2020.

Yi [89] utilized a thiol-ene photoclick strategy to efficiently synthesize multifunctional initiators based on cyclodextrine (CD) cores (Figure 21). The synthesized α-, β-, and γ-CD cores were successfully employed in a “core-first” approach to produce well-defined multiarm star polymers via atom transfer radical polymerization (ATRP), which has been demonstrated as one of the most versatile polymerization techniques for building architecturally complex polymers. Multiarm star polymers are a unique class of branched polymers with a large number of linear arms jointly connected to a central core. Their unique solid and solution properties, due to compact globular architecture and high arm density, make them attractive for a wide range of applications. The author produced the multifunctional core initiator 21BR-S-β-CD by a general procedure of thiol-ene photoclick chemistry, dissolving perallylated β-CD (allyl-β-CD) and DMPA in thiol 2-mercaptoethyl-2-bromo-2-methylpropanoate (HS-EBiB) with a yield of above 90%. In a similar manner, 18Br-S-α-CD and 24Br-S-γ-CD core initiators were synthesized from allyl-α-CD and allyl-γ-CD, respectively, in high yields. Yi also synthesized a new functional thiol 2-mercaptoethyl-2-chloropropanoate (HS-ECP), and in a photoclick reaction with allyl-β-CD, 21Cl-S-β-CD was successfully synthesized. Using 21Br-S-β-CD as the multifunctional core initiator, 21-arm star polymers based on poly(tert-butyl acrylate) (PtBA), polystyrene (PS) and PMMA were successfully prepared. 21-arm poly(N-isopropylacrylamide) (PNIPAM) stars were made from 21Cl-S-β-CD. 18-Arm and 24-arm PtBA stars were produced from 18Br-S-β-CD and 24Br-S-β-CD, respectively. The author expects that the approach could be applied in growing patterned polymer brushes on planar substrates, such as silicon chips and glass slides surfaces.

### 2.5. Polymers of Diazoacetates

Due to the abuse of antibiotics, many treatable illnesses have become incurable. Therefore, bacterial infections caused by drug-resistant bacteria are increasing more and more and the list of (multi)drug-resistant bacteria is becoming longer and longer. As a result, a demand for biomaterials with high potential for suppressing bacterial growth and killing bacteria is increasing tremendously. Diazo compounds are of high interest among researchers, as diazo compounds can act as a precursor for obtaining polymers with polar functional groups. Multiple functional diazo polymers with diazo groups in the backbone and polar functional groups in the side chains can, themselves, be useful materials, and also regarded as templates for predetermined applications after further design and facile modification.

**Figure 22:**
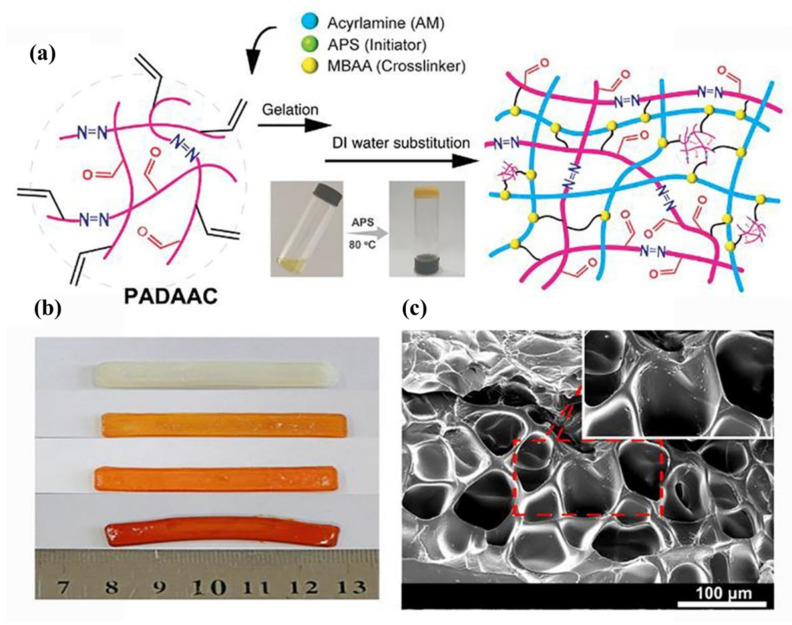
(**a**) Schematic crosslink of PADAAC with AM and MBAA initiated by APS to fabricate hydrogels. (**b**) The color of different contents. (**c**) SEM of lyophilized hydrogel as described by She et al. Adapted with permission from [61]; published by Elsevier, 2020.

She et al. [61] synthesized a unique ternary fluorescent copolymer, poly(allyl diazoacetate-co-acrolein), or (PADAAC), by copolymerization of allyl diazoacetate (ADA) and α,β-unsaturated aldehyde acrolein (AC) in a one-pot, one-step, and catalyst-free reaction (also known as C1N2/C2/C1 copolymerization) for effective bacteria therapy. In this synthesis process, a 1,5-diradical intermediate is formed in the chain initiation step, as a result of fast chain initiation reaction between the double bond of AC and the diradical of ADA. In the chain propagation step, ADA, AC, and CAC (generated from ADA by the loss of N_2_) are inserted. The addition of a hydrogen radical terminates the chain. A brief synthesis procedure was implemented. First, a three-necked round-bottomed flask, equipped with a reflux condensation tube (the other end connected with a safety bottle containing silicone oil to insulate the air), was flushed with Ar gas and charged with ADA in CHCl_3_. Then, the AC solution in CHCl_3_ was incorporated into the flask, drop-wise, through a pressure-equalizing dropping funnel sealed with a glass plug. Subsequently, the polymerization reaction was carried out by heating the reaction mixture at 50 °C with continuous stirring for 8 h. After the polymerization reaction, the polymer products were precipitated in ether and then dried in a vacuum oven to obtain the final product, PADAAC. The PADAAC copolymer contains azo groups in the backbone, which is responsible for its green fluorescent light and contains both allyl and aldehyde groups (at a ratio of 2:1) in the side chain. The authors fabricated a series of acrylamide (AM)-based hydrogels with various amounts of neutral PADAAC as a comonomer with respect to AM monomer by the copolymerization of hydrophilic AM monomer and N, N′-methylene bis(acrylamide) (MBAA) crosslinker (Figure 22). The authors performed post-modification of PADAAC (20 mol%, versus AM monomer) copolymer via a simple imine reaction, where the aldehyde groups of this copolymer were protected with amino groups of 2,2′-(ethylenedioxy)bis(ethylamine) (EBEA). The purpose of this imine reaction is to obtain a hydrogel, whose antimicrobial property can be turned on or off by adjusting the pH.

The authors found that the unusual allyl diazoacetate/acrolein copolymer-based hydrogels with a three-dimensional network structure can suppress bacterial growth and kill the gram-positive Staphylococcus aureus, gram-negative Escherichia coli, and gram-positive methicillin-resistant Staphylococcus aureus, with a killing efficiency of more than 95%. The cytotoxicity of the hydrogels evaluated by the MTT assay was nontoxic towards the fibroblast 3T3-E1 cell. The authors found that the amine-protected PADAAC copolymer displayed no antimicrobial activities on the bacteria due to the hindrance of aldehyde groups; however, the deprotected PADAAC recovered its antimicrobial activities. Furthermore, PADAAC can be readily coated on the glass substrate to get an antimicrobial coating via the solution-immersion coating process. The authors suggested that this new multifunctional PADAAC copolymer with abundant aldehyde and polymerizable allyl-functional groups can be used as a “monomer” to be incorporated into a polymer network or surface coating in any proportion by crosslinking the allyl groups. In addition, the authors considered that the PADAAC copolymer-based hydrogels can be applied as promising antimicrobial agents to combat drug-resistant bacteria in bacteria therapy, and also in many chemical engineering and biomedical fields, as aldehyde functionality is well-recognized for its high chemical activities and bioactivities.

### 2.6. Polystyrene

Polystyrene (plastic products, functionalized polystyrene nanoparticles, fluorescent polystyrene latex beads etc.) has a number of uses in biomedical applications. It is cost-effective, lightweight, transparent, easy to sterilize, and resistant to bacterial growth and moisture. This paved the way for its extensive use in tissue culture trays, diagnostic components, sterile test tubes, petri dishes and other test kits, medical devices, and in medical applications. In this section, we have discussed the synthesis of allyl-functionalized polystyrene polymers and, in some cases, their post-functionalization.

In a study, Zhang et al. [44] prepared well-defined allyl-functionalized telechelic PS and PtBA homopolymers with predetermined molecular weight and narrow polydispersity. RAFT polymerization of styrene was performed using symmetric allyl-functionalized bisallyl trithiocarbonate (BATTC) as a chain transfer agent (CTA) at 110 °C. The polymerization of tert-butyl acrylate (tBA) was achieved using AIBN as an initiator and BATTC as a CTA at 60 °C. The allyl-functionalized BATTC was synthesized from alkyl halides and carbon disulfide in the presence of anion-exchange resin Amberlyst A-26. The authors chose the controlled radical polymerization (CRP) technique over the ionic polymerization, conventional radical polymerization, and polycondensation reactions, mainly to obtain polymers of controlled molecular weight and to perform reactions in the presence of monomers, initiators, or CTA agents containing many functional groups. The authors found that the RAFT thermal polymerization followed first-order kinetics, and the number average molecular weight increased linearly with an increase of monomer conversion (up to ca. 68% conversion).

**Figure 23:**
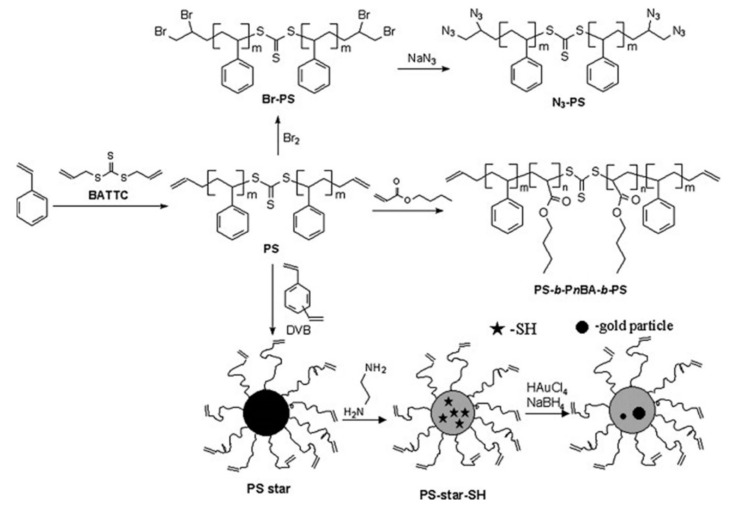
The synthesis route of bisallyl-functionalized telechelic polymers and star polymer and their properties thereof, with BATTC mediated RAFT polymerization as described by Zhang et al. Reprinted with permission from [44]; published by Elsevier, 2006.

To examine the reactivity of the allyl groups of telechelic PS, the authors performed a bromine addition reaction to convert allyl groups into 1,2-dibromopropyl groups, quantitatively. The resultant bromo-terminated PS was further converted to azido-terminated PS [i.e., bis(1,2-diazidopropyl)-capped telechelic PS] by a nucleophilic substitution of the halogen atom. The authors also prepared triblock telechelic copolymers, bisallyl functionalized polystyrene-*b*-poly(n-butyl acrylate)-*b*-polystyrene, (PS-*b*-PnBA-*b*-PS) and poly(tert-butyl acrylate)-*b*-polystyrene-*b*-poly(tert-butyl acrylate), (PtBA-*b*-PS-*b*-PtBA), with the bisallyl functions located at two ends of the triblock copolymers, and star PS with allyl-end-functionalized arms (Figure 23). Later, the star polymer was converted into a difunctionalized star polymer with a thiol-functionalized core by aminolysis reaction using ethylenediamine, which was used as a stabilizer for the formation of gold nanoparticles.

### 2.7. Polyethyleneimine

Polyethyleneimine (PEI) is a synthetic, linear, or branched cationic polymer. It is well recognized for its biocidal potentials, high gene transfection efficiency (referred to as the gold standard for non-viral gene transfection), and ability to build a complex with DNA and proton sponge effect and to facilitate the intracellular transport into nucleus. Due to its exceptional properties, it is used as an attachment promoter (in tissue culture), transfection reagent, permeabilizer of gram-negative bacteria, gene delivery vehicle, and a suitable material for antimicrobial coatings, magnetic NPs coating and targeted therapy (in vitro cell transfection with nucleotides of siRNA or DNA). In this section, we have described the synthesis of allyl functional group containing PEI polymers and, where appropriate, its application.

**Figure 24:**
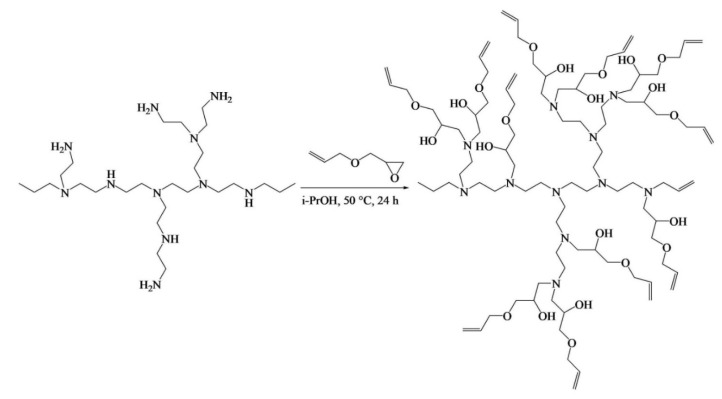
Synthetic route of allyl-terminated hyperbranched polyethyleneimine (PEIene) as depicted by Acebo et al. Adapted with permission from [66]; published by Elsevier, 2016.

Acebo et al. [66] synthesized a new allyl-terminated hyperbranched polyethyleneimine (PEIene) by reacting PEI with AGE in isopropanol solvent, utilizing the benefits of the nucleophilicity of amines of PEI, which react with the oxirane ring (Figure 24).

To cure different formulations of PEIene/diglycidylether of bisphenol A (DGEBA) with pentaerythritol tetrakis (3-mercaptopropionate) (PETMP), the authors [66] performed a two-stage curing process composed of two click reactions: first, a photoinduced thiol–ene addition, followed by a thermal thiol–epoxy reaction. The thiol-ene reaction of allyl functionality is an extremely rapid reaction (completing within a few seconds) and is tolerant to the presence of air/oxygen and moisture. The advantages of allyl groups in multifunctional PEIene are that allyl groups can be readily crosslinked with unsaturated double bond-containing monomers or polymers in a resin formulation, and form a tighter network structure, as the presence of high allyl functionality of hyperbranched polymers increases the glass transition temperature of the product higher than expected. To catalyze both processes, a photoinitiator, DMPA, and a base, 1-methylimidazole (1-MI), were applied, respectively. The authors found that both processes overlapped and the thermal thiol–epoxy process prematurely began during the photoirradiation because the presence of amines in the PEI structure accelerated this process. The thiol–epoxy reaction is accounted for as a simple nucleophilic ring-opening reaction by a thiolate anion formed by H-abstraction with an amine. The alkoxide anion formed is protonated by proton transfer from a quaternary ammonium salt, formed in the activation of thiol or by the thiol itself. Thiol–DGEBA polymerization leads to a network structure with a high molecular weight between crosslinking points.

### 2.8. Allyl Functional Multiene Monomers

**Figure 25:**
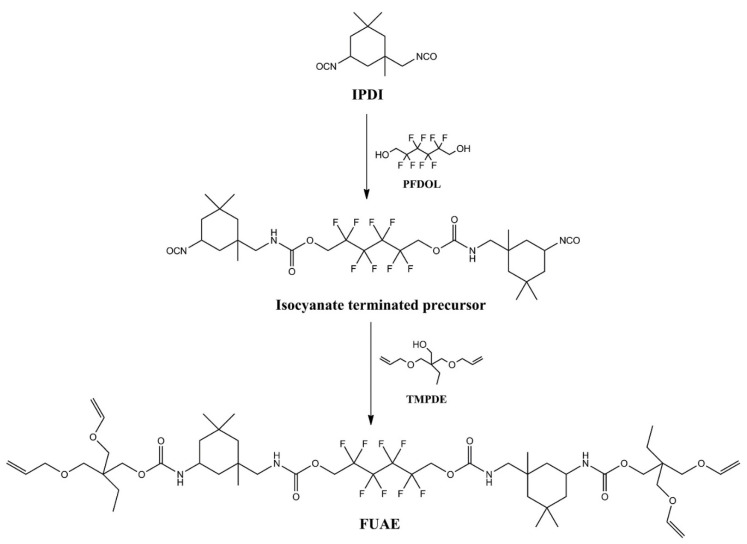
Synthesis route of fluorine containing urethane-based allyl ether multiene monomer (FUAE), as described by Fu et al. Reprinted with permission from [62]; published by Elsevier, 2019.

It is well-known that methacrylate and thiol-ene resin-based composites are commonly used as alternatives to dental amalgams for dental cavity restoration. However, both the methacrylate and thiol-ene systems suffer from significant shortcomings; for example, methacrylate resins show high volumetric shrinkage during photoinitiated polymerization, high polymerization stress, and low functional group conversion. Although thiol-ene systems exhibit a considerable reduction in shrinkage stress due to the delayed gelation, they display significant lower flexural strength and modulus. To solve the problems associated with methacrylate and thiol-ene resin systems, researchers are focused on thiol-ene-methacrylate ternary systems that combine thiol-ene systems with methacrylate systems. Therefore, Fu et al. [62] synthesized fluorine containing urethane-based allyl ether multi-ene monomer (FUAE) and used it to prepare a thiol-ene-methacrylate ternary resin system for dental resin composites with the aim of reducing shrinkage stress. Due to the high viscosity of resins, a reactive diluent, usually triethylene glycol dimethacrylate (TEGDMA), is frequently added to the formulation. To synthesize the FUAE monomer, the authors first synthesized an isocyanate-terminated prepolymer by a condensation reaction of isophorone diisocyanate (IPDI) and 1H,1H,6H,6H-perfluorodecane-1,6-diol (PFDOL) in a stoichiometric ratio of isocyanate and hydroxyl functional groups, using THF and dibutyltin dilaurate (DBTDL) as solvent and catalyst, respectively (Figure 25).

Once the highly reactive isocyanate groups (–N=C=O) reached half of the initial content, the authors added an unsaturated allyl ether, trimethylolpropane diallyl ether (TMPDE) into the reactor, and the reaction was continued until all –N=C=O groups reacted and finally obtained diallyl ether- terminated FAUE monomer as a colorless viscous liquid. The FAUE monomers were mixed with commercial pentaerythritol tetra (3-mercaptopropionate) (PETMA) to prepare a thiol-ene resin system. After that, the thiol-ene resin system was incorporated in a methacrylate-based resin system, 2,2-bis [4-(2-hydroxy-3-methacry-loxyprop-1-oxy)phenyl]propane (Bis-GMA)/TEGDMA, synthesized by Cramer [64] to increase the mass fraction of thiol-ene in the thiol-ene-methacrylate ternary resin system. Camphorquinone photoinitiator (CQ) and 2-dimethylaminoethyl methacrylate co-initiator (DMAEMA) were added into the thiol-ene-methacrylate resin system during the photo-curing process. The ultimate product showed that the presence of FUAE in the ternary resin system increases the degree of conversion of methacrylate functional group and decreases volumetric shrinkage and water sorption and solubility in comparison with neat methacrylate resin-based dental composite. However, the authors conclude that the mass fraction of thiol-ene resin in the ternary resin system must be a maximum of 30 wt% to observe such effects.

**Figure 26:**
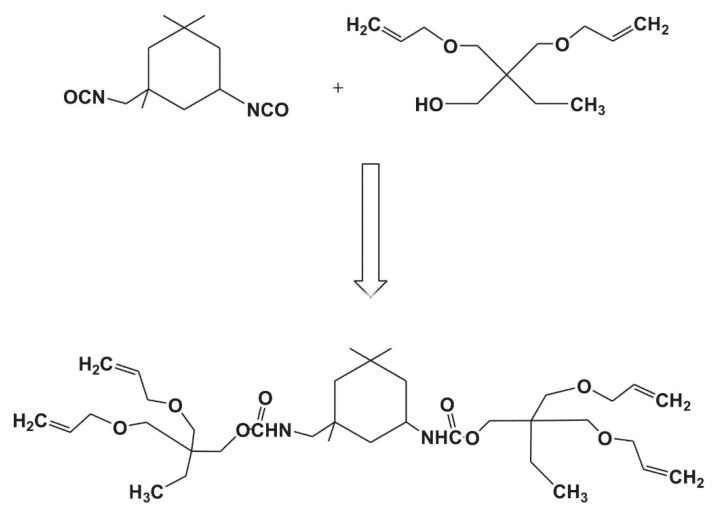
The reaction of isophorone diisocyanate (IPDI) and trimethylolpropane diallyl ether (DAE) for the synthesis of urethane tetra allyl ether monomer (UTAE), as reported by Beigi et al. Reprinted with permission from [63]; published by Elsevier, 2013.

Beigi et al. [63] studied another ternary thiol–ene–methacrylate system as a resin matrix for potential dental restorative composites, synthesizing urethane terta allyl ether monomer (UTAE) as ‘ene’ monomer (Figure 26), and incorporating it into a 2,2-Bis-(2-hydroxy-3-methacryloxypropoxy) phenyl] propane (Bis-GMA)/TEGDMA resin system. The thiol–ene moieties provided lower crosslinked density, more homogeneous microstructure, and displayed higher fracture toughness. The fracture toughness determines the capacity of a dental material to resist brittle fracture and improvement of fracture toughness of a dental material eventually increases longevity and performance. The advantage of allyl functionality in the ternary thiol–ene–methacrylate system is that thiol or ene functional groups react only once with another complementary functional group during thiol–ene step-growth polymerization reaction, which leads to longer spaces between crosslinking sites. In turn, this leads to lower crosslink density, network flexibility, high free volume, and more homogeneity, resulting in higher fracture toughness. The allyl monomer UTAE appears to be promising, but was not explored for any potential application.

Konuray et al. [90] synthesized a set of clickable allyl functional catalytic comonomers for sequential thiol-Michael and radical thiol-ene reactions in preparation of poly(thioether) thermosets. Foix et al. [91] synthesized and characterized a new terminated hyperbranched polyester (HBP), containing thioether and esters in the backbone and hydroxyl groups as chain ends, via an iterative synthetic procedure (a combination of esterification and thiol-ene click reaction), using it successfully as a latent multifunctional macroinitiator for the dual curing of the commercially available bis-cycloaliphatic diepoxide, 3,4-epoxycyclohexylmethyl-30,40-epoxycyclohexyl carboxylate epoxy resin. Flores [92] synthesized and applied an allyl-terminated HBP for the curing of the cycloaliphatic epoxy resin used by Foix [91], 3,4-epoxycyclohexylmethyl-30,40- epoxycyclohexyl carboxylate formulation (a two-stage photoinitiated-thermal dual curing system, consisting of allyl modified hyperbranched polyester and a trithiol compound), and successfully converted the thioether network in a multifunctional thermal macroinitiator to produce a flexible thioether network.

### 2.9. Miscellaneous Polymers

**Figure 27:**
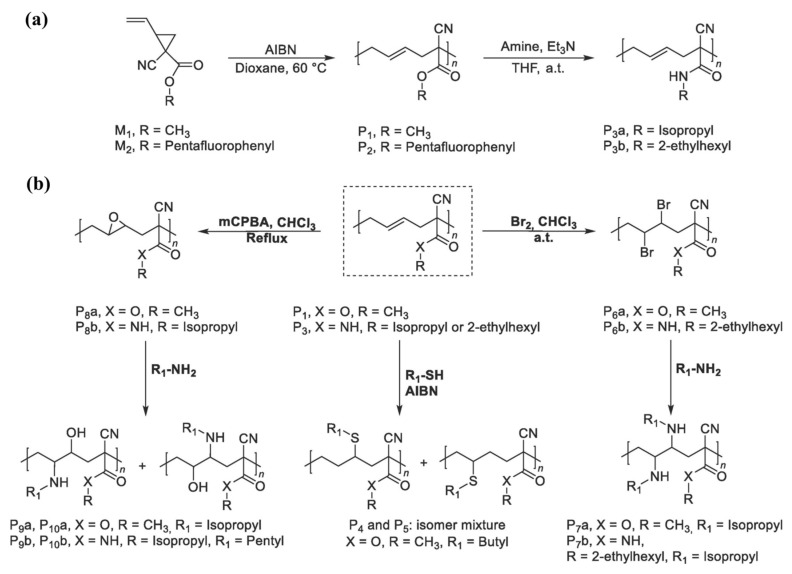
The general scheme presents (**a**): the synthesis of the parent polymers P1, P2, and P3, and (**b**): the bromination, epoxidation, and thiol-ene addition as subsequent post-polymerization modification reactions for the parent polymers, as designed by Ntoukam et al. Reprinted with permission from [93]; published by Elsevier, 2020.

1,1-disubstituted-2-vinylcyclopropane monomers can undergo ROP forming poly(1,1-disubstituted-2-vinylcyclopropanes) that have five carbon atoms and an ethylenic bond in their repeating points. Ntoukam et al. [93] explored the potential of post-polymerization modifications of poly(1,1-disubstituted-2-vinylcyclopropanes). Firstly, they prepared two poly(vinylcyclopropanes) (P1 and P2) with ethylene and ester groups. Aminolysis was performed on activated ester precursor polymer (P2) with isopropyl amine or 2-ethylhexylamine, respectively, giving two readily soluble polymers (P3a and P3b), respectively. Bromination, epoxidation, and thiol-ene addition of the ethylenic moiety were performed as three different functionalization routes. Thiol-ene functionalization was conducted on P1 via a radically driven thiol addition, initiated by AIBN thermal decomposition yielding a polymer product of two regioisomers mixture (P4 and P5) (Figure 27). Furthermore, a ring-opening reaction of the formed oxirane group was performed successfully, yielding polymers P9a and P9b.

## 3. Toxicity Aspect of the “Allyl” Functional Polymers

Kost et al. [58] evaluated that the blank NPs prepared from the tGA and ACC-functionalized copolymers experienced no cytotoxicity against all tested cell lines (normal L929, HeLa, and AGA cell lines) in the MTT assay after 24 h of incubation at 37 °C. Incubation of MDA-MB-231 cells with mPEG-*b*-PJL-OH polymer showed that more than 90% of cells were found to be viable after 48 h of incubation time for polymer concentrations up to 2 mg/mL, in the study by Bansal et al. [59]. Le Devedec et al. [60] investigated the cytotoxicities of the crosslinked polymer matrices (CP_39K_ and CP_15K_) prepared from PVL-*co*-PAVL copolymers against L929 mouse fibroblast cells by the extraction dilution method. Both CP_39K_ and CP_15K_ cylindrical matrices exhibited excellent in vitro biocompatibility (more than 80% L929 cell viability evaluated using the MTS assay) at all extract dilution percentages (100, 50, 25, 12.5, 6.25, and 3.125%) and time points considered for the experiment (24, 48, and 72 h). Liang et al. [75] reported that, after 48 h incubation, the free nanocarrier, PEG-HBPAE-OH unimolecular micelles, exhibited nearly no cytotoxicity against HeLa cells, even at high polymer concentration (1.0 µg/mL) in the MTT assay. The toxicity levels of HAP-poly(AMA-COOH) nanohybrids were evaluated against HeLa cells and A549 cells at three concentrations of nanohybrids (0.1, 0.5, and 1 mg/mL) for 24, 48, and 72 h using MTT assay by Bach et al. [76]. The results revealed that about 80% of cells (both HeLa and A549 cells) were found to be viable, even after 72 h of incubation with 0.1 mg/mL nanohybrids concentration. Though the percentage of cell viability decreased with the increase in nanohybrids concentration, nonetheless, above 60% of cells were still measured to be viable at a nanohybrids concentration of 1 mg/mL. Lu et al. [51] reported that the mPEG-*b*-PAGE-*b*-PCL micelles showed excellent biocompatibility and low cytotoxicity to both human oral epidermoid carcinoma (KB) and human gastric carcinoma (SGC) cell lines. The authors assessed the cytotoxicity of the blank micelles against KB and SGC cell lines using MTT assay, and found that the cell viability after 48 h incubation was more than 90%, at a micelles concentration of 0.1 mg/mL. Su et al. [37] determined the cytotoxicity of PDMS-*g*-APEG_2400_-PHMG to human aorta smooth muscle cells (SMCs) by the MTT and LIVE/DEAD cell viability assays. The authors observed no significant difference in the cell viability among cells cultured with PDMS-*g*-APEG_2400_-PHMG, pristine PDMS, and on tissue culture polystyrene plates in the MTT assay. They also found that PDMS-*g*-APEG_2400_-PHMG and pristine PDMS had no effect on the cell growth for up to 5 d in the LIVE/DEAD assay. Ding et al. [88] evaluated the in vitro cytotoxicity of pH-responsive UV-crosslinkable C_6_ O-allyl chitosan (OAL-CS) solution (injectable hydrogel, with concentration 0.1–1.0 mg/mL) and of the hydrogel extract. The cell counting kit-8 (CCK-8) assay of L929 fibroblast cells was used in this experiment. The authors observed that more than 95% relative cell viabilities were accounted for in all OAL-CS solutions (concentration 0.1–1.0 mg/mL within 24 h and 48 h) and more than 100% relative cell viabilities were calculated for by the hydrogel sample extract. She et al. [61] evaluated the cytotoxicity of unusual allyl diazoacetate/acrolein copolymer-based hydrogels by MTT assay. All the hydrogels were found to be nontoxic to the 3T3-E1 cell. The cell viability of the tested hydrogels was calculated to be more than 75%; for the amine-protected hydrogel, viability reached above 85%.

The important toxicity aspect of poly(CL-AGE) [40], PEG-*b*-P(LA-*co*-AGE) [36], PGlCL [77], poly(ε-allyl-ε-caprolactone) [78], poly(ester-anhydride)s [48,50], polycarbonates [80], poly(1,1-disubstituted-2-vinylcyclopropanes) [93], multiarm star polymers [89], bisallyl-functionalized telechelic polymers and star polymer [44], PEIene [66], and urethane-based allyl ether multi-ene monomers [62,63], which are discussed in this study, has not yet been evaluated, or at least not reported, in the reviewed articles.

## 4. Conclusions and Outlook

This review summarizes recent advances in the synthesis of allyl-terminated polymers, including post-modifications and applications, especially in the biomedical field. Alongside conventional polycondensation reactions, ROPs, C1N2/C2/C1 polymerization, and reversible addition-fragmentation chain transfer (RAFT) polymerization methods are now performed to synthesize allyl polymers or macromonomers. Several allyl-terminated polymers reported in this study have demonstrated promising results towards their biomedical applicability, especially in drug delivery and dental applications. Allyl-functionalized polymers, whose synthesis and post-modification techniques are only described, but their potential application not yet explored, can be investigated further to understand their utility in drug delivery and tissue engineering. Nevertheless, not all the allyl-terminated polymers or polymers functionalized with allyl groups described in Section 2 are explored in biomedicine. Some polymers were tested for other technical applications, whereas the applications of other potential polymers have not yet been explored. Thus, there is still much room for future tailored approaches of polymers with “allyl” functionality within the pharmaceutical and biomedical fields.

## Figures and Tables

**Table 1:** Characteristics of the PVL-*co*-PAVL copolymers as studied by Le Devedec et al. Adapted with permission from [60]; published by American Chemical Society, 2018.

M_n_ (g/mol)								
PVL-*co*-PAVL *^a^*	GPC *^b^*	PDI	^1^H NMR *^c^*	NB Allyl Groups *^d^*	% AVL *^e^*	T_m_ (°C) *^f^*	Δ°H_m_ (J/g) *^g^*	χ_c_ (%)
P_7_._5K_	9300	1.45	7500	15	28	12.6	36	25
P_15K_	12,000	1.45	15,000	25	23	34/39.3	76	53
P_32K_	24,000	1.47	32,000	45	20	38.2	58	40
P_39K_	33,000	1.52	39,000	25	9	41.6/47.6	88	61

*^a^* P_7_._5K_, P_15K_, P_32K_, and P_39K_ refer to the different PVL-*co*-PAVL copolymers. *^b^* Number average molecular weight (g/mol) obtained from GPC analysis. *^c^* Number average molecular weight (g/mol) obtained by ^1^H NMR spectroscopy. *^d^* Number and *^e^* weight percentage (% M.Wt) of allyl valerolactone in the copolymer (% AVL) based on the total molecular weight determined by ^1^H NMR spectroscopy. *^f^* Melting temperatures (Tm) and *^g^* enthalpy of melting (H_m_) and degree of crystallinity χ_c_ (%) were determined by DSC analysis (2nd cycle).

**Table 2:** Minimum inhibitory concentration (MIC) of AGE and APEG modified PHMG oligomers, as studied by Su et al. Reprinted with permission from [37]; published by Wiley, 2017.

MIC [µg mL^−1^]	Gram-Negative (G−)	Gram-Positive (G+)	Fungus
	*P. aeruginosa*	*S. aureus*	*F. solani*
PHMG	5.0	2.5	2.5
A-PHMG	5.0	2.5	2.5
APEG_1200_-PHMG	5.0	5.0	5.0
APEG_2400_-PHMG	10.0	10.0	10.0
